# Community perceptions of paediatric severe anaemia in Uganda

**DOI:** 10.1371/journal.pone.0209476

**Published:** 2019-01-03

**Authors:** Aggrey Dhabangi, Richard Idro, Chandy C. John, Walter H. Dzik, Godfrey E. Siu, Robert O. Opoka, Florence Ayebare, Michael B. van Hensbroek

**Affiliations:** 1 Child Health and Development Centre, Makerere University College of Health Sciences, Kampala, Uganda; 2 Department of Paediatrics and Child Health, Makerere University College of Health Sciences, Kampala, Uganda; 3 Ryan White Centre for Paediatric Infectious Disease and Global Health, Indiana University School of Medicine, Indianapolis, IN, United States of America; 4 Department of Pathology (Transfusion), Harvard University / Massachusetts General Hospital, Boston, MA, United States of America; 5 Department of Global Child Health, Emma Children's Hospital, Academic Medical Centre, University of Amsterdam, Amsterdam, the Netherlands; University of Saskatchewan, CANADA

## Abstract

**Background:**

Severe anaemia remains a major cause of morbidity and mortality among children in sub-Saharan Africa. There is limited research on the beliefs and knowledge for paediatric severe anaemia in the region. The effect of these local beliefs and knowledge on the healthcare seeking of paediatric severe anaemia remains unknown.

**Objective:**

To describe community perceptions of paediatric severe anaemia in Uganda.

**Methods:**

Sixteen in-depth interviews of caregivers of children treated for severe anaemia and six focus group discussions of community members were conducted in three regions of Uganda between October and November 2017.

**Results:**

There was no common local name used to describe paediatric severe anaemia, but the disease was understood in context as ‘having no blood’. Severe anaemia was identified to be a serious disease and the majority felt blood transfusion was the ideal treatment, but concomitant use of traditional and home remedies was also widespread. Participants articulated signs of severe pediatric anemia, such as palmar, conjunctival, and tongue pallor. Other signs described included jaundice, splenomegaly, difficulty in breathing and poor appetite. Poor feeding, malaria, splenomegaly and evil spirits were perceived to be the common causes of severe anaemia. Other causes included: human immunodeficiency virus (HIV), haemoglobinuria, fever, witchcraft, mosquito bites, and sickle cell. Splenomegaly and jaundice were perceived to be both signs and causes of severe anaemia. Severe anaemia was interpreted to be caused by evil spirits if it was either recurrent, led to sudden death, or manifested with cold extremities.

**Conclusion:**

The community in Uganda perceived paediatric severe anaemia as a serious disease. Their understanding of the signs and perceived causes of severe anaemia to a large extent aligned with known clinical signs and biological causes. Belief in evil spirits persists and may be one obstacle to seeking timely medical care for paediatric severe anaemia.

## Introduction

Severe anaemia is a common health problem among children, accounting for 10–29% of all paediatric admissions [1–3] and about 8–17% of hospital deaths in sub-Saharan Africa [1,4]. In Uganda, 11% of children under five years are anaemic [5]. The biological causes of paediatric severe anaemia in sub-Saharan Africa have been widely documented as being complex and multi-factorial, involving malaria, micronutrient deficiencies, viral agents and sickle cell anaemia [6–8]. However, the perception, which encompasses knowledge and beliefs of paediatric severe anaemia among local African communities, has received little attention, and therefore a limited understanding of how local perceptions are related to clinical diagnoses and how local beliefs influence treatment seeking behaviours exists.

There is evidence to suggest that community perception of disease or illness and their interpretation (including the names used to describe it), are closely linked to supernatural, societal and biological elements [9]. Anthropologist Arthur Kleinman has presented a theoretical model for understanding health care systems derived from the way people react to sickness in local social and cultural settings as well as how they perceive, label or name, explain, and treat sickness [10]. According to Kleinman, people’s health beliefs and behaviours are governed by cultural rules and systems. Thus, culture establishes general criteria that guide the therapeutic process and how people evaluate the outcomes. Our study of local perceptions of severe anaemia draws insight from this model. We find it informative in exploring the cultural beliefs and attitudes of the community about severe anaemia and its causes and how these aspects might influence their health seeking behaviours. This study therefore explored the perceptions of pediatric severe anaemia, including knowledge about its presentation, perceived cause, and treatment among caregivers as well as local community members in three regions of Uganda.

## Materials and methods

### Study design

This was a qualitative study of community perceptions of paediatric severe anaemia in Uganda that used in-depth interviews (IDIs) as the primary instrument to gather data complemented by focus group discussions (FGDs) [11]. This study was nested in an on-going clinical trial on post-discharge malaria chemoprevention among children recently treated for severe anaemia (NCT02671175). Interviews were conducted between October and November 2017.

### Study area

This study was conducted in regional referral hospitals in three of the ten regions of Uganda, namely *Buganda*, *Busoga* and *Bunyoro* in the districts of the *Greater Masaka (central-)*, *Jinja (eastern-)* and *Hoima (mid-western Uganda)*, respectively. Different regions were investigated to accommodate the cultural diversity of Uganda.

### In-depth interviews

A total of 16 in-depth interviews were held with parents or guardians (caregivers) of children recently treated for severe anaemia (within three months) or who were, at the time, admitted to hospital for treatment of severe anaemia. The interviewees were purposively selected to include men and women caregivers, grandparents and young parents, and care-givers of children who had experienced recurrent severe anaemia or died from severe anaemia. We interviewed five, six, and five respondents at Masaka, Jinja and Hoima regional referral hospitals, respectively. Saturation was achieved by the final interviews at each site, and no new information was gained [12]. Potential participants were either invited by phone call to come for the interview at the hospital, using records of the on-going clinical trial described above, or identified from among those still in the ward. Caregivers of children known to have sickle cell anaemia or cancer were excluded. The topics explored during the interview included local names for anaemia and their meaning, perceived causes, signs and symptoms, prevention and care seeking for severe anaemia, including blood transfusion. Training of research assistants was conducted during which data collection tools were translated into the local languages prior to data collection. Interviews were conducted in the local languages by experienced qualitative researchers, lasting about 40 minutes each and audio-recorded.

### Focus group discussions

A total of six (two per district) focus group discussions (FGDs) of six to eight members each were conducted. Participants of FGDs included only mothers from local communities (within 20 km radius) of the respective regional referral hospitals, mobilized and hosted by a Village Health Team (VHT) member. The communities (villages) were purposively selected from among those frequently visited by the social workers during follow up of study children on the on-going clinical trial described above. The VHT member was given clear instructions regarding whom to invite; namely, mothers of child bearing age, living in the same locality, and known to each other. The mobilized groups were further screened by the researchers to exclude elderly women, and young girls. After formal introductions, participants were initially briefed about the study goals and procedures, as well setting ground rules before discussions commenced. An FGD guide was used to explore topics similar to those of the IDIs. For each region, FGDs were conducted after IDIs, in order to follow up on any emerging themes with community members, such as stigma. A trained moderator conversant with the respective local languages facilitated the discussions that lasted 50 to 70 minutes each, while an assistant audio-recorded the conversation and noted non-verbal communications.

### Data analysis

Data analysis followed the thematic approach, which seeks to identify patterns within the data that make up a coherent account, which can best interpret or explain a particular phenomenon [13, 14]. A debrief meeting preceded data transcription and analysis work. The research assistants transcribed the audio recorded data verbatim and translated them into English. The first author (AD) attended all IDI and FGD sessions and took field notes. He checked the transcripts for completeness and accuracy before they were uploaded into ATLAS.ti Version 7.5.12 software. Based on Arthur Kleinman’s (1975) model of health care system, code books were developed using deductive codes from the interview and focus group discussion topic guides as well as inductive codes that emerged from the data after reading a sample of six transcripts. Four authors (AD, FA, GES, and MBvH) contributed to code book development. Once the code books were finalized, coding was undertaken by FA and code reports were generated centrally and shared with co-authors for review. Code summaries were then developed and the findings were synthesized thematically.

### Ethical approval

The study was reviewed by the Research and Ethics Committee of Makerere University School of Medicine, and cleared by the Uganda National Council for Science and Technology. Written informed consents were obtained from all IDIs and verbal consent from FGD participants.

## Results

### Interviewee characteristics

The demographic characteristics of in-depth interviewees are summarized in Table 1. All of the 16 interviewees were rural residents, and had limited education but were familiar with caring for a child with severe anaemia. All, except one, cared for more than one child. Two respondents were single mothers, and seven respondents’ children had experienced recurrent episodes of severe anaemia. The median age of caregivers (IQR) was 36 (30–44) years.

**Table 1:** Characteristics of caregivers of children with severe anaemia interviewed (n = 16).

Variable		Frequency (%)
Caregiver’s relationship with child	Biological parent	12 (75%)
	Grand parent	4 (25%)
Gender of caregiver	Female	12 (75%)
	Male	4 (25%)
Occupation of caregiver	Subsistence farmer	15 (94%)
	Nursery teacher	1 (6%)

n = sample size.

### Overview of themes

Four broad themes regarding paediatric severe anaemia were identified from the analysis, namely; the local name for severe anaemia, its causes, the signs of severe anaemia, and its treatment and prevention. We found that severe anaemia had no common name by which it is referred to locally. The disease was attributed to multiple causes, and medical pluralism is practiced in its treatment. With few exceptions, we observed no major differences across the regions of *Buganda*, *Busoga* and *Bunyoro* in terms of general understanding of severe anaemia, its perceived causes, or health care seeking behaviours by the local community members.

### Severe anaemia in context

We asked caregivers of children to describe the most common diseases their children (under five years of age) suffered from in the recent past, in order to contextualise severe anaemia. Respondents indicated that malaria, cough, and flu were the common childhood diseases encountered at their homes. Although not reported as common, severe anaemia was identified to be an uncompromising illness and a major cause for concern among caregivers of children:

“*Mostly it is cough* …*When they are playing with their colleagues who may be suffering from cough*, *they also contract it”* (IDI 01_Hoima). *“They (children) are commonly disturbed by cough*, *only Melisa (grand-daughter) is the one who got severe anaemia and it brought us challenges here in the hospital*. *That is the only serious case I have seen among my children”* (IDI 04_ Hoima). “*Whenever a child has severe anaemia*, *all you think about next*, *is death”* (Respondent [R]-2_FGD1_ Masaka).

### Local name for severe anaemia

Across the three regions, interviewees referred to severe anaemia literally as ‘loss of blood’; ‘having no blood’; or ‘being blood deficient’. Apart from these literal phrases, there were no specific terms used by locals in reference to severe anaemia. There were no further clues despite probing for the local name of the disease:

*“We still refer to it as loss of blood*. *I think the terminology remains the same*, *[that the child has lost blood]”* (IDI 01_ Masaka). *“Me I have never heard of a local name…it is possible that there are people who know it but for us we do not know…*.*”* (Respondent [R]-01_FGD1_ Jinja).

On three occasions, three FGD participants used the terminology “*musana”*, *“obwayi*” and “*kamuli*”, respectively to describe severe anaemia. However, these terms were unanimously rejected by fellow community members during discussions as misleading since they meant different diseases. The FGD participants clarified that the first two terms refer to severe acute malnutrition in *Buganda* and *Busoga* regions respectively, while the latter is a local name for neonatal jaundice in *Buganda* region. In Masaka, for example, FGD participants objected to these names:

“*No*, *that [kamuli as the name for severe anaemia] is not right”*. *“A child is born with kamuli*.*”* (R3&5 FGD1_ Masaka). *“A child with kamuli does not get severe anaemia*, *but the entire body turns yellow*. *It comes shortly after the child is born*, *and the first thing they (health workers) test for is kamuli*, *and it sometimes kills*, *leaving the child’s corpse yellow”* (R4_ FGD1_ Masaka).

In the *Busoga* community, children who experience recurrent episodes of severe anaemia were labelled or nick-named as “*kanywa musaayi*”, which means “the one who just drinks the blood”. This label tends to assign blame on the sick child for not retaining the blood transfused into him/her, and instead easily loses it as though he/she were just drinking it.

Despite this label, children who suffered recurrent severe anaemia—rather than being stigmatized as is the case with many chronic diseases—are regarded with a sense of empathy by community members:

“*They are called kanywa musaayi; those who get a blood transfusion*....*Yes*, *because he suffered from severe anaemia and got a blood transfusion*....*he would have drunk it (blood) from the hospital…”* (IDI 04_ Jinja). “*You look at how the child looks and feel so sad for him*, *we only advise him to go to the hospital and we do not segregate him*, *because it can as well attack your relative”* (R7 & R4_FGD2_Masaka).

### Recognition of signs and symptoms of severe anaemia among children

The signs of severe anaemia among children were well articulated and appeared to be fairly well understood by both interviewees and FGD respondents who described them with ease. Generally, the signs commonly mentioned included bodily changes, mainly of the eyes, face, feet or legs, and the palm or hands. Specifically, conjunctival pallor or jaundice, feet or facial oedema, and paleness of the palms and the tongue were common signs for severe anaemia reported by many respondents:

“*I also want to share*,*…when my child gets severe anaemia; the feet*, *hands and the eyes become* pale, *the face turns yellow*. *He becomes weak and even fails to sit*” (R4_FGD2_Hoima).

Other signs included: swelling of the abdomen, difficulty in breathing, poor appetite, fevers, and hair changes. Whether swelling of the feet (pitting pedal oedema) occurs in anaemic children was debated by participants, with some arguing that it only occurs in pregnant women. The pedal oedema sign and the loss of the sub-cutaneous fat with resulting loose skin, not only provoked debate but also seemed confusing, resulting in misclassification of severe anaemia and severe acute malnutrition:

“*It is usually the feet of pregnant women that get swollen”*. *“No*, *even the feet of children get swollen once they get severe anaemia*. *When you touch them*, *you feel a depression in their feet”* (R5 & R2_FGD1_Masaka).

Another sign of paediatric severe anaemia was the presence of cold extremities (cold feet with hot trunk). This sign was not only understood to mean severe disease among participants, but also was perceived to be associated with either evil spirits or splenomegaly as etiological agents, as illustrated below:

“*Yes; because the child abruptly gets hot up (trunk)*, *and cold down (feet)*, …*that one (sign)*, *even when you do not understand*....*you would discover later* …*”* (IDI 06_ Jinja). “*The child becomes very hot but the legs will be cold as though they have been soaked in water* …*That is how you can tell that a child has an enlarged spleen”* (R3_FGD1_Masaka).

Splenomegaly itself was also described as a sign of severe anaemia:

“*When a child has severe anaemia*, *there is something that swells here…*.(She touches the left-upper part of her abdomen to show the exact part that swells*)*. *Whenever that thing attacks*, *the child loses the blood”* (R4_FGD2_Hoima).

Regarding the symptoms of paediatric severe anaemia, mothers were quick to note that most children with severe aneamia may be too young, not able to talk yet, and thus would not report any symptoms. However, a few symptoms that interviewees said their children with severe anaemia might report included malaise, dizziness, and anorexia:

“*A child might be young and has not started talking yet*, *but you observe for yourself”* (R3_FGD1_Hoima). “*For the grown-ups*, *they will feel unwell and very weak*. *They say; I do not have strength and I feel my body is cold”* (IDI 02_ Masaka).

### Perceived causes of severe anaemia in children

Severe anaemia was perceived to be caused by either a disease process such as malaria, poor feeding, an enlarged spleen (splenomegaly), evil spirits or a blood sucking agent, and a list several other causes. To describe the process or mechanisms of severe anaemia, local terms that refer to ‘sucking blood’ such as in the case with evil spirits or ‘draining the blood out of the child’, such as in the case of splenomegaly were used interchangeably. In particular, respondents described five main causes of severe anaemia, which we describe below:

#### Poor feeding

The common view amongst both community members and caregivers was that poor feeding was a leading cause of paediatric severe anaemia. Their interpretation of poor feeding was not eating a balanced diet, eating cold food, such as leftovers, and eating ‘dirty food’, all of which were attributed to parental negligence. Indeed, some care-givers assigned the blame to themselves for the poor care of their children, while others attributed severe anaemia to poverty:

“*Poor care*,*… giving food that does not build blood*. *Giving one type of food; not changing diet for the child”* (IDI 02_ Hoima). “*Poor feeding is when the child does not eat food that supplement blood in the body*, *for example green vegetables* (“*enva endirwa”*) (IDI 03_ Masaka). “*Even poverty causes severe anaemia”* (R5_FGD2_Masaka). *“The way he used to feed when he was staying with his mother was different from the way I am feeding him now*. *So I think that it could be the poor feeding; because since his childhood he had never suffered from severe anaemia—that was the first time*.*” “She (child’s mother) brought him to the village (to me) and the situation changed*. *I have many children; I cannot manage to fulfil the way he was feeding”* (IDI 02_ Jinja).

Despite these interpretations, community members did not clearly understand the mechanisms by which poor feeding causes severe anaemia:

“*God created our bodies and put blood in it* …*but this body is there because of the foods that he prepared for it*. *When one of those foods misses*, *the blood reduces”* (IDI 04_ Hoima).

#### Diseases perceived to cause severe anaemia

The majority of community members believed that there is always an underlying disease that causes severe anaemia. Malaria was described as the leading cause of severe anaemia among children:

“*Can a child have severe anaemia without any sickness*?*” “The fact is*, *a child must first get sick which later results into severe anaemia*. *I cannot get severe anaemia from nowhere;* …*that cannot happen*. *It must be brought about by a disease*”. (R2 & R3—FGD1_Masaka). “*I think malaria is the most common; because it is very strong and once it attacks a child*, *it affects him/her badly”* (IDI 04_ Masaka). “*I think that it could be malaria* …*because it makes the body hot and the blood gets finished* …*”* (IDI 02_ Jinja). “*Because when she gets malaria*, *the yellowing of the eyes sets in*, *then it turns to the blood”*(IDI 02_ Jinja).

Malaria was believed to cause severe anaemia through several mechanisms; through high fevers which were believed to dry up the blood, if malaria was severe, poorly treated or manifested with convulsions:

*“Every time the body gets so hot*, *the blood goes because it moves very fast”* (R1 & R6_FGD1_Jinja). “*The failure to finish the dose*, *…you take the medicine but do not give it to the child well*. *The child would have been poorly treated*, *and the malaria remains in the body and affects him/her*. *So the more you poorly treat the malaria*, *the more it sucks blood from the child”* (R4_FGD1_Masaka).*“There are some children whose blood vessels get blocked due to convulsions*, *when they fall sick so when the blood vessels get blocked and there is no blood circulating*....*it causes severe anaemia*.*”* (R5_FGD2_Jinja). With the exception of one participant who described clues of possible synergy between malaria, jaundice (‘*enkaaka’*), and splenomegaly, the majority did not understand the mechanisms of severe anaemia in malaria.

The other diseases that were believed to cause severe anaemia were HIV/AIDS and typhoid:

“*AIDS is the worst of all*, *in sucking blood*. *There is a neighbor’s child*. *She was born with HIV but most of the time she was anaemic*. *She eats but does not grow fat*” (R6_FGD2-_Masaka).

#### Splenomegaly

Splenomegaly, in addition to being perceived as a sign of severe anaemia, was a dreaded cause of severe anaemia that was perceived as draining blood out of a child even to death, if not treated. These beliefs were found in all three regions, but were strongest in *Buganda*, followed by *Busoga*. The local terms used to describe splenomegaly were; ‘*ekikubuuko’*, ‘*akabengo’* and ‘*ekibaale*’ among the *Baganda*, *Basoga* and *Banyoro* tribes respectively.

In the *Buganda* region, all six mothers and four out of seven in our first and second FGDs respectively had taken their children to an herbalist for the treatment of splenomegaly, where they cut on the skin over the abdomen:

“*It sucks a lot of blood*”. “*Yes it does; because when you take the child to be cut (by the herbalist)*, *the child does not bleed and the reason you will be given for this*, *is that it had already sucked the child’s blood”* (R2 & R3_FGD1_Masaka).

Most community members believed that an enlarged spleen sucks blood directly from the heart, and that is caused by either malaria or high fever, but one elderly interviewee explained it to be caused by breast milk spilling from the mother’s opposite breast onto the baby’s abdomen during nursing. In addition, community members assigned gender to splenomegaly, depending on the size and its response to local treatment. They believed an enlarged spleen was likely to be ‘female’ if located in the lower part of the abdomen (large) and if it responded quickly to local treatment; while a ‘male one’ is located in the upper part, and poorly responds to treatment:

“*They say it is brought about by fever; that state when the child is very hot*” (R2_FGD1-_Masaka). “*I hear the “male enlarged spleen” (‘akasajja’) rests at the upper part of the abdomen while the female one (‘akakazzi’) on the lower part of the abdomen”* (R1_FGD1-_Masaka).

#### Evil spirits

Community members did not think evil spirits, often referred to as the African traditional disease (‘*ebyekiddugavu’*), were a common cause of severe anaemia. Nevertheless, evil spirits were suspected to be the cause of severe anaemia if: the child does not improve on formal treatment and blood transfusion, if no cause for the severe anaemia was readily known or not communicated to them, if child with severe anaemia died suddenly, if severe anaemia became recurrent or when one of the signs on the child was a temperature difference between the torso and legs, as illustrated by the following quotes:

*“This is the difference; the child who has severe anaemia as a result of malaria*, *when taken to hospital and they receive a blood transfusion*, *they get what*? …*well*, …*but the one attacked by ‘bitega’ (in chorus) does not get well”* (R2_FGD1_Jinja). “*Sometimes even when the child is taken to hospital*, *they test and they do not find the cause of sickness but child keeps loosing blood*, *which confirms that there are spirits sucking blood from that child*” (R4_FGD1_Jinja).

Evil spirits were categorized into four groups: ‘*amayembe’* (demons), ‘*ekitega’* (another kind of demon), ‘*misambwa’* (fallen angels), and ‘*magiini’* (coastal spirits that manifest as humans). ‘*Ekiteg*a’ were regarded as the most efficient at sucking blood. They were believed to be sent by witch-doctors to suck blood, and come in the form of wind, or use certain animals like lizards or snakes as agents to carry them:

“Yes....*majority of them (spirits) are just sent* …*and whenever you see a thing such as a snake or lizard*, *just know there is something behind it”* (Chorus_FGD1_Jinja).

Issues related to evil spirits causing severe anaemia were particularly prominent among two parents (IDIs), whose belief in evil spirits had far reaching social consequences; one believed the spirits killed her child, and the other shifted from his ancestral home to another district as a mitigation measure against spirits:

“*I avoided it by selling my land where I was residing and bought land in another place*. *She (the witch) reached the extent of praising herself that she wanted to finish my family*. *So I left for her the village*, *and now she is a goddess of that village”* (IDI 06_ Jinja).

### Other causes of severe anaemia

Several other causes of paediatric severe anaemia were explained including witchcraft (locally called *‘ebihara’* in *Bunyoro)*, bloody diarrhoea, mosquitoes, haemoglobinuria, ‘*false teeth’* (incipient canine teeth), and sickle cell anaemia (termed as ‘*Nalubiri’* in *Buganda*). Some community members also understood jaundice (locally termed as “*enkaaka*”) to be a disease entity that causes severe anaemia, rather than being a sign; while others referred to jaundice as ‘yellow fever’, which is a completely different disease:

“*When mosquitoes bite her*, *they suck blood; don’t they*?” (IDI 01_Hoima). “*Sometimes he urinates blood*, *and every time he urinates blood*, *the blood gets finished*” (IDI 02_ Jinja). “*They say false teeth* (‘*ebiinyo’*, as they are locally called) *also suck blood*, *and when you do not remove them* (germectomy), *the child dies”* (R8_FGD1_Hoima).

### Seeking healthcare, treatment, and prevention of severe anaemia

From the interviews, biomedical treatment of severe anaemia in health facilities was held in high regard, with blood transfusion, as often indicated, highly acceptable. However, participants also reported using traditional and home remedies, particularly after discharge from hospital, or even modifying the biomedical advice, suggesting a mixed appreciation of medical systems in these communities. Blood building syrups, green leafy vegetables such as beet-root (*Beta vulgaris)*, Scarlet egg-plant (*Solanum aethiopicum shum*), *Ammaranthus species* and other local herbs were used for this purpose:

“*I bring the child to you; the doctors who are experts to see what you can do for him/her*” (IDI 04_Masaka). “*We bring them to hospital…*. *they are tested by taking blood from them to find out the blood group which they have to give them*. *Thereafter*, *they put blood in them”* (IDI 01_Masaka). “*When I get back home*, *I give the child green vegetables (‘enva endirwa’)*, *milk and silver fish to help her restore more blood*,.” (IDI 05_ Masaka).

Two divergent discourses emerged concerning paediatric severe anaemia prevention practices. Many interviewees said they did nothing to prevent severe anaemia when the children were not ill. In contrast, some reported that they would feed their children ‘well’ and give them blood building herbs:

*“If the child is healthy and not sick*, *I also do not bother*, …*I do not trouble myself cooking local herbs”* (R3 & R5_ FGD1_ Masaka). “*Before he gets sick*?… …*I do not do anything; The child first gets sick then you can think of what to do*, …*will you treat what you have not seen*?*”* (IDI 04_ Jinja). “*We make sure that we feed them well and we also give them the local herbs even when they are not sick”* (R2_ FGD1_ Masaka).

## Discussion

This study used in-depth interviews of primary caregivers of children treated for severe anaemia and complemented the IDIs with FGDs with mothers from the community to study local perceptions of childhood severe anaemia in Uganda. Because perceptions influence health care-seeking behaviours, a better understanding of the knowledge and beliefs regarding severe anaemia is important for effective community-based health interventions. We explored the local names for severe anaemia, what caregivers considered important signs/symptoms, and the perceived severity of the disease and its causes. The results of our study suggest that the community’s understanding of severe anaemia to a large extent align with known clinical signs and biological causes. However, the existence of negative local beliefs, such as those in evil spirits can potentially undermine the benefits of such local knowledge and hinder appropriate healthcare seeking for paediatric severe anaemia in Uganda.

Although severe anaemia is a relatively common and well-known disease among children, our findings suggest that in select central, eastern and western communities of Uganda, there is no common local name for severe anaemia; rather the disease is understood in context, as ‘loss of blood’, ‘lack of blood’ or ‘having no blood’. In many cultures, diseases are named and the names given usually convey people’s interpretation and definitions. For example, among the Luo speaking communities in northern Uganda cervical cancer is named “*two remo*”referring to an illness that manifests with bleeding [15], and in some ancient European societies, epilepsy was named “*morbus sacer*” referring to a demoniac condition [16]. The lack of a specific term for severe anaemia may be a result of inadequate vocabulary in many local languages. Having no name by which a disease is known by the local community may have negative consequences for the management of that disease by lay people. In the current study we found a mis-classification of severe anaemia as severe acute malnutrition. This was possibly due to the absence of a specific name to describe severe anaemia. Another possible explanation for this finding of having no name might be related to the revelation that community members perceived severe anaemia to be a consequence of another underlying condition, such as poor feeding or malaria. Indeed, apart from genetic disorders and primary bone marrow failure syndromes, most anaemias are secondary to other causes.

The local understanding of signs and symptoms of a range of childhood illnesses have been reported; for example, many studies have evaluated how the community recognizes symptoms of malaria, and have mainly focused on convulsions and fever [17–19] but no study has evaluated severe anaemia, even though it is the leading complication of childhood malaria. In this study, we found that community members were able to articulate the signs of severe anaemia, and their definitions included the same signs used in clinical settings, such as pallor of the palms, conjunctiva, and tongue [20–23]. They also ably explained many other signs, such as jaundice and difficulty in breathing. There was, however, some tendency to confuse some signs of severe anaemia with severe acute malnutrition. Indeed this may even occur in clinical settings, where co-existence of severe anaemia and severe acute malnutrition requires strict protocol adherence in their management [24]. Similarly, jaundice (locally termed as ‘*enkaaka*’) was misunderstood to be a disease entity that causes severe anaemia. The tendency to misclassify diseases by local community members calls for attention when designing community child-health programmes.

In most rural African communities, illnesses tend to be attributed to social and supernatural causes. Social aetiologies include beliefs in witchcraft and sorcery [25]. In some cases, local perceptions of an illness may be complex, such that social, supernatural, individual, and natural or biological causes are ascribed to it. We found that attributable causal explanations for paediatric severe anaemia by community members were largely related to poor feeding, malaria, and splenomegaly, with locally constructed beliefs in evil spirits causing severe anaemia being heightened under certain circumstances, such as recurrent severe anaemia and sudden death. Previous studies examining perceptions of disease causation in Africa have reported that evil spirits, and splenomegaly are perceived to cause many diseases, such as febrile seizures [26]. In Uganda, local beliefs about splenomegaly and its local treatment using therapeutic cuts around the splenic surface are widespread [17]. In Ethiopia, abortion, prolonged labour and epilepsy have been attributed to evil spirits [27]; while in a South African study, traditional healers believed evil spirits caused epilepsy [28]. Despite these observations, the majority of factors perceived by the community to cause severe anaemia in children align with documented biological causes [6–8]. Such substantial insight into the causes and understanding of the physical signs of severe anaemia, are great opportunities to enhance community health education. Blood transfusion can be a life-saving treatment for children with severe anaemia. Our findings suggest that most caregivers of children with severe anaemia would seek treatment from hospitals, and that blood transfusion was acceptable. We found many knowledge gaps about prevention of paediatric severe anaemia, with most community members expressing the opinion that no action was needed to protect ‘their healthy children’.

### Limitations

The study design omitted interviews from opinion makers and cultural leaders, whose views might be insightful. However, the views of such individuals are one-step removed from those with primary responsibility for the care of children with severe anaemia. This study mainly focused on mothers and recruited 75% interviewees and 100% FGDs as mothers; because they are most often the primary caregivers of children. However, in a typical African setting, fathers who were less represented here also make many of the health care decisions [29].

### Recommendations

The Ugandan policy and public health workers need to pay attention to how community perceptions about severe anaemia influence caregivers’ response to this condition, and design appropriate messages on early detection, appropriate treatment and prevention. In addition, community sensitization addressing misperceptions such as those related to splenomegaly, jaundice and local beliefs in evil spirits represent important targets for improvement in paediatric severe anaemia care.

## Supporting information

S1 Code Reports (RTF)
