# Supplementary material for: Community perceptions of paediatric severe anaemia in Uganda
Source: PLoS One. 2019 Jan 3;14(1):e0209476. doi: 10.1371/journal.pone.0209476 (PMC6317806; doi:10.1371/journal.pone.0209476)
Supplement: S1 Code Reports — (RTF) [file pone.0209476.s001.rtf]

Code Family: FGD 1.0 Local names for SA
______________________________________________________________________

HU:	Local Perceptions of Childhood Severe Anaemia_FA_January 2018
File:	 [C:\Users\Florence\Desktop\Local Perceptions of Childhood Severe Anaemia_FA_January 2018.hpr7]
Edited by:	Super
Date/Time:	2018-01-05 09:53:10
______________________________________________________________________

Created: 2017-12-18 09:42:58 (Super) 
Codes (8):	[10.1 BT fears] [10.2 BT safety concerns] [FGD 1.1 Local name for SA] [FGD 1.2 Meaning of local name for SA] [FGD 1.3 Local name for recurrent SA] [FGD 1.4 meaning of local name for SA] [FGD 1.5 RSA general perception] [FGD 1.6 Labels given to children with RSA]
Quotation(s): 37
______________________________________________________________________

10.1 BT fears
______________________________________________________________________

P 2: IDI_Severe_Anaemia_Jinja_002.rtf - 2:18 [Interviewers: (mhmn) You menti..]  (226:233)   (Super)
Codes:	[10.1 BT fears - Families (2): BT_fears and concerns, FGD 1.0 Local names for SA] 
No memos

Interviewers: (mhmn) You mentioned something about the blood affecting the child.....
Respondent: eeeh (yes) 
Interviewer: How?
Respondent: people from the community often say those things " imwe mwena (you also) every time a child is sick that you take him to get a blood transfusion. Do you know that sometimes they put bisayi (bad blood) that will affect him and you can even lose the child"...but me i told them that if he is to die, that will be God's will. Even those who don't get a blood transfusion die. I have to take him and they put blood.
Interviewer: What causes the effect?
Respondent: I don't know
Investigator: How do this bad blood affect the child? what does it contain, why do they refer to it as bisayi (bad blood).
Respondent: Its them who know........

P 4: IDI_Severe_Anaemia_Jinja_004.rtf - 4:15 [Interviewer: Now putting aside..]  (187:190)   (Super)
Codes:	[10.1 BT fears - Families (2): BT_fears and concerns, FGD 1.0 Local names for SA] 
No memos

Interviewer: Now putting aside what you think........we want you to tell us what people say...that when a child undergoes a blood transfusion..heee! don't do this, don't do that..what do they say?
Respondent: Some say that when they put a child on blood transfusion, your child may not survive. Others say that when they get blood transfusion, you may be lucky and the child regains. Others say that children who gets blood transfusion are  very difficult to take care of because once transfused, it means that each time they get sick, they will require a blood transfusion. So if he does not get blood transfusion, he will not survive
Interviewer: eehehee.. that once transfused then they will always require blood transfusion all the time....
Respondent: Yes.

P 6: IDI_Severe_Anaemia_Jinja_006.rtf - 6:14 [Interviewer: Now, when the chi..]  (189:196)   (Super)
Codes:	[10.1 BT fears - Families (2): BT_fears and concerns, FGD 1.0 Local names for SA] 
No memos

Interviewer: Now, when the child is undergoing transfusion, do have anything that goes through you mind? May be this or that, it could be a worry…fear …
Respondent: For me I may not fear…..
Interviewers…what about others?…who fears?
Respondent: Some people fear that they migh give them….mhmhm…the blood that has “our ka thing”…….mhmhm.
Interviewer: which “ka thing”?......
Respondent: mhmhm…our ka thing = 'ka HIV'……..so some people have doubts about that…..
Interviewer: that the blood could have the the HIV virus.........
Respondent: mmm…(yes)……..

P 7: IDI_Severe_Anaemia_Masaka_001.rtf - 7:18 [F: We've already looked at tha..]  (193:195)   (Super)
Codes:	[10.1 BT fears - Families (2): BT_fears and concerns, FGD 1.0 Local names for SA] 
No memos

F: We've already looked at that, we now need your opinion about the blood transfusion treatment.
F: What are your fears about blood transfusion?
R: In case I find that there is no blood in the hospital, I get afraid that my child might die.

P 7: IDI_Severe_Anaemia_Masaka_001.rtf - 7:22 [F: It seems you don't move aro..]  (208:209)   (Super)
Codes:	[10.1 BT fears - Families (2): BT_fears and concerns, FGD 1.0 Local names for SA] 
No memos

F: It seems you don't move around or talk with other people.
R: They say that the blood being transfused to the child might be having the HIV virus.

P 8: IDI_Severe_Anaemia_Masaka_002.rtf - 8:27 [F: You said you used to fear b..]  (220:223)   (Super)
Codes:	[10.1 BT fears - Families (2): BT_fears and concerns, FGD 1.0 Local names for SA] 
No memos

F: You said you used to fear blood transfusion?
R: Yes, I feared that the blood could be infected with the HIV virus.
F: Is there any other thing that was making you afraid of?
R: No, it's only the HIV virus

P 9: IDI_Severe_Anaemia_Masaka_003.rtf - 9:18 [F: Have you faced any fear or ..]  (150:155)   (Super)
Codes:	[10.1 BT fears - Families (2): BT_fears and concerns, FGD 1.0 Local names for SA] 
No memos

F: Have you faced any fear or worry about blood transfusion before?
R: I'm worried that children who will have received a blood transfusion earlier will always lose blood each time they get sick. 
F: What else?
R: the other thing is that the blood transfused to the child may be having the HIV virus because this blood is sometimes got from a person who is HIV positive.
F: What else?
R: That's all.

P10: IDI_Severe_Anaemia_Masaka_004.rtf - 10:20 [F: You mean there is nothing y..]  (194:195)   (Super)
Codes:	[10.1 BT fears - Families (2): BT_fears and concerns, FGD 1.0 Local names for SA] 
No memos

F: You mean there is nothing you fear about blood transfusion?
R: As long as I know that it is going to save the life of my child then there is nothing to fear because fearing will not bring about any solution for my child. When I bring the child to hospital all I want is his life to be saved.

P11: IDI_Severe_Anaemia_Masaka_005.rtf - 11:15 [F: What are your fears? R: I f..]  (174:177)   (Super)
Codes:	[10.1 BT fears - Families (2): BT_fears and concerns, FGD 1.0 Local names for SA] 
No memos

F: What are your fears?
R: I fear only one thing and I always speak it our even if my child is not sick and this is about the safety of the blood being transfused. Is this blood good or could it be that they have got it from an HIV positive person and is transfused to those with severe anemia. That is the only thing that I fear.
F: What do you think?
R: I don't know whether this blood is got from an HIV positive or negative person?

P13: IDI_Severe_Anaemia_Hoima_002.rtf - 13:14 [SA; Now do you have any concer..]  (170:175)   (Super)
Codes:	[10.1 BT fears - Families (2): BT_fears and concerns, FGD 1.0 Local names for SA] 
No memos

SA; Now do you have any concerns or fears about blood transfusion in children
RES; Yes, I feared at first…mmm... They would tell me if they do blood transfusion in a child that she can't live long...mmm...
SA; is it the only one you used to fear?
RES; That the child becomes small and also loses her understanding / intelligence.
SA; I haven't understood that.
RES; Now let's say the child was understanding well, and everything…mmm….but after blood transfusion, somehow she starts not to understand well, ok she can't come back to her original understanding

P14: IDI_Severe_Anaemia_Hoima_003.rtf - 14:17 [SA; OK. Do you have any fears ..]  (178:181)   (Super)
Codes:	[10.1 BT fears - Families (2): BT_fears and concerns, FGD 1.0 Local names for SA] 
No memos

SA; OK. Do you have any fears or concerns about blood transfusion in children?
RES; according to me I can't fear because I know that when they put blood on him he is going to get well. The only concern can be on care after; not to be exposed to sunshine, not to be exposed to coldness, I become concerned about his life because he has been given blood which was not created in him.
SA; Uhhh... How?
RES; let's say you're of blood group A and they give you O because O serves all; there they are not yet compatible with the already existing blood. so I have to protect him well. I cover him well during coldness and during hot temperatures. I sit him properly under the shade and make sure am caring for him well. I fell afraid about that thing…uhhh..

P19: FGD_Severe_Anaemia_Jinja_01.rtf - 19:46 [the thought i will have will b..]  (456:456)   (Super)
Codes:	[10.1 BT fears - Families (2): BT_fears and concerns, FGD 1.0 Local names for SA] 
No memos

 the thought i will have will be immediately after the blood transfusion, my child might die.....that is what i was going to say....you have brought your child, they have put the blood and you are asking yourself, will there be a difference when that transfusion is complete? there is taking the child and they put the fist bottle and the second bottle and there is no change . .....they tell you to add another, they give him the full dose of tablets but immediately you reach home, the child dies.....

P19: FGD_Severe_Anaemia_Jinja_01.rtf - 19:47 [Respondents: may be when you a..]  (466:466)   (Super)
Codes:	[10.1 BT fears - Families (2): BT_fears and concerns, FGD 1.0 Local names for SA] 
No memos

Respondents: may be when you are not sick....at this time you fear because your child is not sick but when you are sick because there is taking you when you are in a critical condition....how will you fear  a blood transfusion?......another issue is that you might have been getting a blood transfusion but which ever child gets a transfusion...that one dies,....mama so and so, the child has severe anemia and she is under transfusion. when they come back home, the child dies...that care giver will always be skeptical because every time they take the child to the hospital, the child dies. It is like the blood transfusion bids the child bye....that is what worries her most.

P19: FGD_Severe_Anaemia_Jinja_01.rtf - 19:48 [Respondent: Me as a care giver..]  (474:474)   (Super)
Codes:	[10.1 BT fears - Families (2): BT_fears and concerns, FGD 1.0 Local names for SA] [10.2 BT safety concerns - Families (2): BT_fears and concerns, FGD 1.0 Local names for SA] 
No memos

Respondent: Me as a care giver if i have ever lost a child because of that.......even when she has never lost a child.........even when you have never lost a child for example at the moment there is AIDS, some people are not sure of that blood and that is what worries me as a parent......that they will give them blood that has HIV.....some people know their blood group....

P19: FGD_Severe_Anaemia_Jinja_01.rtf - 19:49 [Respondents: (in chorus)...tha..]  (478:478)   (Super)
Codes:	[10.1 BT fears - Families (2): BT_fears and concerns, FGD 1.0 Local names for SA] 
No memos

Respondents: (in chorus)...that may be the blood has HIV ....they put on my child and after one year.....some of the health workers are bad hearted these days they inject themselves....after one year and i take back the child then they tell me the child has HIV when i and the father don't have......and they will have taken a prior test when he does not have and the second time, they tell you he has....some healthy workers infect the children...it has ever happened and people talk about it......was it in kampala?.....that health worker the care giver took the child for treatment and it was on news that she injected herself when she was infected and injected someone's child.....they tested the child was sick and the health worker was sick.....so you have those fears with that blood that is why we boil our herbs and we don't go for medical treatment....majority of people fear that blood.....say we fear!.......we real fear it (blood) hahaha......

10.2 BT safety concerns
______________________________________________________________________

P19: FGD_Severe_Anaemia_Jinja_01.rtf - 19:48 [Respondent: Me as a care giver..]  (474:474)   (Super)
Codes:	[10.1 BT fears - Families (2): BT_fears and concerns, FGD 1.0 Local names for SA] [10.2 BT safety concerns - Families (2): BT_fears and concerns, FGD 1.0 Local names for SA] 
No memos

Respondent: Me as a care giver if i have ever lost a child because of that.......even when she has never lost a child.........even when you have never lost a child for example at the moment there is AIDS, some people are not sure of that blood and that is what worries me as a parent......that they will give them blood that has HIV.....some people know their blood group....

FGD 1.1 Local name for SA
______________________________________________________________________

P17: FGD_Severe_Anaemia_Hoima_01.rtf - 17:1 [Now how do you call severe ane..]  (25:26)   (Super)
Codes:	[FGD 1.1 Local name for SA - Family: FGD 1.0 Local names for SA] 
No memos

Now how do you call severe anemia among children in runyoro language?
Res; That's how we say it; “the child has lost blood” (omwana kuhwebwamu omusayi)..mmm…

P17: FGD_Severe_Anaemia_Hoima_01.rtf - 17:2 [R1; even doctors at the hospit..]  (29:31)   (Super)
Codes:	[FGD 1.1 Local name for SA - Family: FGD 1.0 Local names for SA] 
No memos

R1; even doctors at the hospital they tell us the child has lost blood (omwanan ahweirwemu omusayi) so he needs blood transfusion.
SA; Uhhhh. Is there any other person with a different name?
Res; No. 

P18: FGD_Severe_Anaemia_Hoima_02.rtf - 18:1 [SA; How do you call severe ane..]  (27:30)   (Super)
Codes:	[FGD 1.1 Local name for SA - Family: FGD 1.0 Local names for SA] 
No memos

SA; How do you call severe anemia in your language (runyoro)?
Res; We say “the child has lost blood”.( ahweiremu omusayi)
SA; What else?
Res; child doesn't have blood (Omwana taina musayi)

P19: FGD_Severe_Anaemia_Jinja_01.rtf - 19:1 [Here in Butiki, how is severe ..]  (29:32)   (Super)
Codes:	[FGD 1.1 Local name for SA - Family: FGD 1.0 Local names for SA] 
No memos

Here in Butiki, how is severe anemia called in the local language (lusoga)……the name, how is severe anemia called in the local language?....
Respondents: (in agreement) That is what we know………that a child has no blood (severe anemia)
Moderator: There is no name for it?.....……
Respondents: No…we just say that “the child has no blood” (severe anemia)

P20: FGD_Severe_Anaemia_Jinja_02.rtf - 20:1 [Respondent: Severe anemia? Mod..]  (18:28)   (Super)
Codes:	[FGD 1.1 Local name for SA - Family: FGD 1.0 Local names for SA] 
No memos

Respondent: Severe anemia?
Moderator: yes....when a child has severe anemia...does that disease have a name?
Respondent:  me i think Obwayi..............Obwayi.....
Moderators: Obwayi or  what does another person have to say?.......Obwayi?..... is that severe anemia?
Respondents: Yes, its kwashiorkor....... 
Moderators :Mmnm.....we are not talking about  Kwashiorkor, we mean severe anemia in children….. does it have a name in Lusoga?
Respondents: (in chorus) me think...i thought Obwayi is Kwashiorkor......because when a child gets malnourished...........
Moderator: Obwayi is Kwashiorkor?...., ahaaaa.......
 Respondents: The child has no blood........ahaha..when the child has no blood they say anemic......that is english....
Moderator: okay now in Lusoga.......
Respondent:....but in Lusoga?

P20: FGD_Severe_Anaemia_Jinja_02.rtf - 20:2 [Moderator: obwayi is kwashakor..]  (35:36)   (Super)
Codes:	[FGD 1.1 Local name for SA - Family: FGD 1.0 Local names for SA] 
No memos

Moderator: obwayi is kwashakor.......but what is severe anemia?
Respondents: ............... “omwana agheiremu Omusayi” (the child has no blood) 

P20: FGD_Severe_Anaemia_Jinja_02.rtf - 20:5 [Moderator: ok..... ok so we ha..]  (58:59)   (Super)
Codes:	[FGD 1.1 Local name for SA - Family: FGD 1.0 Local names for SA] 
No memos

Moderator: ok..... ok so we have agreed that we are not sure what lumuli means and that we (moderators) shall try to find out what Lumuli is......lets say we don't know but we shall ask those typical basoga
Moderator: we have agreed that we are not sure of how its called 

P21: FGD_Severe_Anaemia_Masaka_01.rtf - 21:1 [F: What do you call severe ane..]  (61:68)   (Super)
Codes:	[FGD 1.1 Local name for SA - Family: FGD 1.0 Local names for SA] 
No memos

F: What do you call severe anemia in your language?
R4: Its name doesn't change either, we also say that the child has had a severe lack of blood. You can detect this through the child's body turning pale
F: We shall be looking at the signs later. What local name is given to the severe lack of blood?
All Rs: The name is the same, severe lack of blood
F: Don't you have any other name for it
R1: You mean a “Luganda” one apart from English
R5: it is still referred to as “severe anemia” whether it is caused by poor feeding or any other thing.
R3: blood will have been lost

P22: FGD_Severe_Anaemia_Masaka_02.rtf - 22:1 [F: Ok… (All laugh)...Now I wis..]  (41:44)   (Super)
Codes:	[FGD 1.1 Local name for SA - Family: FGD 1.0 Local names for SA] 
No memos

F: Ok… (All laugh)...Now I wish to first know how you call severe anemia in your language.
R3: Kamuli (becomes yellow in color)
F: They call it “kamuli?”
R2: Mmm.. And she turns to yellow color.

P22: FGD_Severe_Anaemia_Masaka_02.rtf - 22:2 [F: The disease is called kamul..]  (47:48)   (Super)
Codes:	[FGD 1.1 Local name for SA - Family: FGD 1.0 Local names for SA] 
No memos

F: The disease is called kamuli? 
R5: Mmm.. which means she becomes yellow…mmmm.. And swells….

FGD 1.2 Meaning of local name for SA
______________________________________________________________________

FGD 1.3 Local name for recurrent SA
______________________________________________________________________

P17: FGD_Severe_Anaemia_Hoima_01.rtf - 17:7 [SA; now I wish to ask you. The..]  (46:47)   (Super)
Codes:	[FGD 1.3 Local name for recurrent SA - Family: FGD 1.0 Local names for SA] 
No memos

SA; now I wish to ask you. There are some children who gets recurrent severe anemia. How do you call that in runyoro?
R1; It is “losing blood every time (kuhwebwamu omusayi burikaire)..mmmm… and the truth is if the child is like two months I can't know whether he has severe anemia unless I take him to the doctor and confirms to me.

P18: FGD_Severe_Anaemia_Hoima_02.rtf - 18:6 [SA; Now the name of that thing..]  (56:57)   (Super)
Codes:	[FGD 1.3 Local name for recurrent SA - Family: FGD 1.0 Local names for SA] 
No memos

SA; Now the name of that thing in runyoro
R6; It is “kugarukanizamu”(Recurrent)..mmm.. it takes like one week and it comes back..mmm..  and there is another disease that also disturbs children

P19: FGD_Severe_Anaemia_Jinja_01.rtf - 19:4 [Moderator: Now, if there is no..]  (53:54)   (Super)
Codes:	[FGD 1.3 Local name for recurrent SA - Family: FGD 1.0 Local names for SA] 
No memos

Moderator: Now, if there is no name given to severe anemia, how about if a child gets severe anemia often may be for example every after a month….when they get recurrent episodes of severe anemia…….how is that called? Does it have a specific name?
Respondents (in agreement) No…we don't have any difference…..they will still just….

P19: FGD_Severe_Anaemia_Jinja_01.rtf - 19:5 [Moderator: mhmhmn……it has no s..]  (56:56)   (Super)
Codes:	[FGD 1.3 Local name for recurrent SA - Family: FGD 1.0 Local names for SA] 
No memos

Moderator: mhmhmn……it has no specific name .................?

P19: FGD_Severe_Anaemia_Jinja_01.rtf - 19:6 [Respondent: But that does not ..]  (59:59)   (Super)
Codes:	[FGD 1.3 Local name for recurrent SA - Family: FGD 1.0 Local names for SA] 
No memos

Respondent: But that does not have a name….it doesn't…..it could be that the name is there but we don't know it….but they can call him/her 'Kamusayi' is there again she has severe anemia….ka “lukozimba”……the child….but even when you are a big person they will say again she is there mbu she has severe anemia…she is a lukozimba her life is not well all the time……

P19: FGD_Severe_Anaemia_Jinja_01.rtf - 19:9 [Moderator: But the disease has..]  (72:73)   (Super)
Codes:	[FGD 1.3 Local name for recurrent SA - Family: FGD 1.0 Local names for SA] 
No memos

Moderator: But the disease has no name?
Respondents: me I have never heard of a name…it could be possible that there people who know it but for us we don't know…..

P20: FGD_Severe_Anaemia_Jinja_02.rtf - 20:4 [Moderator: how about....there ..]  (40:49)   (Super)
Codes:	[FGD 1.3 Local name for recurrent SA - Family: FGD 1.0 Local names for SA] 
No memos

Moderator: how about....there are those that suffer from severe anemia from time to time...many times...(recurrent SA)...this week severe anemia....next time severe anemia......does it have a name or it doesn't?........ 
Respondents: .......that one we have the name
Moderator: ....you have it........So what do you call it?
Respondents: we have a name.........
Moderators: how do you call it? that disease that.....
Respondents: that child...what ...... that one we call the child has sickle cells..... 
Moderator: no... no we shall come to that, ....first wait ......
Respondents: (inchorus) the situation where a child suffers severe anemia all the time......what is the disease called?.....not the child.....but the diseases........mmmm.....how it is called......
Moderator: You might.......yeah tell us....do you lable the child too? 
Respondent: No....not the child...i just gave an example you can have a child who often suffers severe anemia.......what about what they call olumuli....what is it? .....what is that disease called?

P21: FGD_Severe_Anaemia_Masaka_01.rtf - 21:6 [F: What do you call recurrent ..]  (98:101)   (Super)
Codes:	[FGD 1.3 Local name for recurrent SA - Family: FGD 1.0 Local names for SA] 
No memos

F: What do you call recurrent severe anemia in your language?
R3: are you referring to the situation where the child loses blood each time?
F: Yes
R1: Some say that that child has an enlarged spleen (ekikubuuko) which drinks blood 

P21: FGD_Severe_Anaemia_Masaka_01.rtf - 21:7 [F: We are coming to that, we w..]  (102:106)   (Super)
Codes:	[FGD 1.3 Local name for recurrent SA - Family: FGD 1.0 Local names for SA] 
No memos

F: We are coming to that, we want to first know the name given to recurrent severe anemia?
R2: We have failed to answer that, we don't know the name given to that situation.
R1: The time I was at Mulago referral hospital I discovered it' name which I can't remember now.
F: You mean you forgot it on your way back?
R2: We have failed to answer that one

P22: FGD_Severe_Anaemia_Masaka_02.rtf - 22:3 [F: There are children who expe..]  (49:52)   (Super)
Codes:	[FGD 1.3 Local name for recurrent SA - Family: FGD 1.0 Local names for SA] 
No memos

F: There are children who experience recurrent severe anemia. How do you call that in your language?
R4: It is caused by poor feeding.
F: I am not asking for the cause; I am asking how you call recurrent anemia in children.
R3: It's the same disease, the child fails to walk (omwana butatambula)

FGD 1.4 meaning of local name for SA
______________________________________________________________________

P19: FGD_Severe_Anaemia_Jinja_01.rtf - 19:7 [Moderator: “Lukozimba”…….what ..]  (60:61)   (Super)
Codes:	[FGD 1.4 meaning of local name for SA - Family: FGD 1.0 Local names for SA] 
No memos

Moderator: “Lukozimba”…….what does that mean?
Respondents: A child who is never healthy and never happy and there is no time that the child will be happy………all the time the child is weak….

FGD 1.5 RSA general perception
______________________________________________________________________

FGD 1.6 Labels given to children with RSA
______________________________________________________________________

P19: FGD_Severe_Anaemia_Jinja_01.rtf - 19:8 [Moderator: What about saying “..]  (66:70)   (Super)
Codes:	[FGD 1.6 Labels given to children with RSA - Family: FGD 1.0 Local names for SA] 
No memos

Moderator: What about saying “kamusayi”, ........what does it mean?
Respondents: (all) “every time the child is getting a blood transfusion”…..all the time the child is being given blood…all the time……
Moderators: But those names are given to the person not the sickness….
Respondents: mmmh?……yes....
Moderator: we would like to know the difference, the name is given to the person who is sick but not the disease……….


Code Family: FGD 2.0 Signs of SA
______________________________________________________________________

HU:	Local Perceptions of Childhood Severe Anaemia_FA_January 2018
File:	 [C:\Users\Florence\Desktop\Local Perceptions of Childhood Severe Anaemia_FA_January 2018.hpr7]
Edited by:	Super
Date/Time:	2018-01-05 09:57:09
______________________________________________________________________

Created: 2017-12-18 09:43:16 (Super) 
Codes (2):	[FGD 2.1 Signs] [FGD 2.2 Symptoms]
Quotation(s): 19
______________________________________________________________________

FGD 2.1 Signs
______________________________________________________________________

P17: FGD_Severe_Anaemia_Hoima_01.rtf - 17:3 [SA; What are the signs of seve..]  (32:34)   (Super)
Codes:	[FGD 2.1 Signs - Family: FGD 2.0 Signs of SA] 
No memos

SA; What are the signs of severe anemia among children?
R2; for me if my child has severe anemia, the face changes and the body turns to yellow color and when I check the eyes they turn pale and that child becomes dizzy…mmm… if he is walking you see him experiencing dizziness…mmmmm….
R3; for me if my child gets severe anemia….mmm… She becomes yellow, the face and the feet swell..mmm…

P17: FGD_Severe_Anaemia_Hoima_01.rtf - 17:4 [SA; another one? R1; if my chi..]  (35:38)   (Super)
Codes:	[FGD 2.1 Signs - Family: FGD 2.0 Signs of SA] 
No memos

SA; another one?
R1; if my child gets severe anemia all eyes become pure white (pale), the abdomen swells and the whole body becomes pale. Even the finger nails become pale….without anything like red.
SA; Any other thought?
R4. I also want to share… when my child is experiencing severe anemia, the feet become pale, arms and the eyes, the face turns to yellow. He becomes weak and even fails to sit. 

P17: FGD_Severe_Anaemia_Hoima_01.rtf - 17:5 [R7; if my child is experiencin..]  (39:40)   (Super)
Codes:	[FGD 2.1 Signs - Family: FGD 2.0 Signs of SA] 
No memos

R7; if my child is experiencing severe anemia, the eyes swell and he wants all the time to be on sunlight.
R5; when my child gets severe anemia…mmm…the feet swell and has no appetite for food.

P18: FGD_Severe_Anaemia_Hoima_02.rtf - 18:2 [SA; now how can you know that ..]  (31:38)   (Super)
Codes:	[FGD 2.1 Signs - Family: FGD 2.0 Signs of SA] 
No memos

SA; now how can you know that your child has severe anemia
R1; for me I can know when my child keeps becoming sick, not having appetite for food, when he loses weight,…uhhh… the child can become weak and the palms starts becoming pale. And you get to know that the child has no blood due to fever.
SA; we are looking at the signs of severe anemia among children
R3; for me my child's eyes becomes too pale (white), and becomes yellow on the face, uhhh… doesn't have appetite and gets persistent fevers.
R2; when the child has severe anemia she becomes yellow on the face and palms and sometimes the body changes
SA; How?
R2; She all turns yellow….. and there you get to know that the child is experiencing severe anemia.
R4: when a child has severe anemia there is something that swells here..(She touches the leftside part of her abdomen to show the exact part that swells). When that thing attacks, she loses blood.

P18: FGD_Severe_Anaemia_Hoima_02.rtf - 18:3 [SA; which thing is that? R4; I..]  (39:44)   (Super)
Codes:	[FGD 2.1 Signs - Family: FGD 2.0 Signs of SA] [FGD 4.4 Enlarged spleen - Family: FGD 4.0 Causes of SA] 
No memos

SA; which thing is that?
R4; I dint know it but they say its stonelike (we call it ekibare)……referring to an enlarged spleen….
SA; which side?
Res; This side (…. As some of the respondents touch their left part of abdomen to show where it swells from…) for it doesn't come outside but swells from inside.
SA; Uhh…what else?
R4; She feels stomach ache.

P18: FGD_Severe_Anaemia_Hoima_02.rtf - 18:4 [SA; What else? R4; The whole f..]  (45:48)   (Super)
Codes:	[FGD 2.1 Signs - Family: FGD 2.0 Signs of SA] 
No memos

SA; What else?
R4; The whole face changes even if she is in darkness you can see her. Even shows no blood in the finger nails…mmmm..
SA; How does the face become?
R4; It all becomes yellow…mmm…

P19: FGD_Severe_Anaemia_Jinja_01.rtf - 19:2 [Moderator: As care givers, wha..]  (33:38)   (Super)
Codes:	[FGD 2.1 Signs - Family: FGD 2.0 Signs of SA] 
No memos

Moderator: As care givers, what signs do we see and we know that the child has got severe anemia?
Respondents: The eyes become pale…… the child becomes pale….
Moderator: Let us talk one at a time
Respondents: The child becomes pale…..
Moderator: How does the child become pale?
Respondents: The child becomes pale and the eyes become white and they say that the child has no blood (severe anemia)

P19: FGD_Severe_Anaemia_Jinja_01.rtf - 19:3 [Moderator: Mhmnhm? Matha… Resp..]  (39:42)   (Super)
Codes:	[FGD 2.1 Signs - Family: FGD 2.0 Signs of SA] 
No memos

Moderator: Mhmnhm? Matha…
Respondent: Most of the time the eyes become pale, secondly, the child can seem like swelling the face, thirdly, some children's hair changes the colour
Moderators: mmmm…but if you say that the hair changes colour, you have to tell us from which colour to which colour…..
Respondents: It changes to yellow or deep brown…..but another thing is that the child can swell whether the feet and when you press any part of his feet, it forms sort of a hole (= “pitting edema”) (.........all in agreement)

P20: FGD_Severe_Anaemia_Jinja_02.rtf - 20:3 [Respondents: (in chorus) the c..]  (37:37)   (Super)
Codes:	[FGD 2.1 Signs - Family: FGD 2.0 Signs of SA] 
No memos

Respondents: (in chorus) the child gets pale...when they get severe anemia......still it is called severe anemia.....in my view...because the child with severe anemia can be pale....mmmm......

P20: FGD_Severe_Anaemia_Jinja_02.rtf - 20:6 [Respondents: t....the eyes bec..]  (62:71)   (Super)
Codes:	[FGD 2.1 Signs - Family: FGD 2.0 Signs of SA] 
No memos

Respondents: t....the eyes become pale and the palms are so pale
Moderator: .......hands..... how?...here (pointing in the palms) 
Respondent: mmm.....on the palm and they become pale......
Moderator: mmhm....... what else?
Respondents: You can also tell when the child has lost appetite and weight.......another thing is that you can know from the colour of urine ......mhm?.... its so so so yellowish 
Moderator: mhmmmm....it (urine) turns yellow?
Respondents: because yellowing sucks the blood out of a child.....the urine is so so yellow......yellow....the urine......
Moderator: mmm....what next?
Respondents: (in chorus) .....the ffet swell, ....ahaaa....the feet swell.......even the eyes swell..... and the child becomes very cold and even the cheeks swell abnormally......even mine whenever he could get severe anemia....the ankles swell...all the feets swell and the cheeks swell abnormally...
Moderator: mmmhm......

P21: FGD_Severe_Anaemia_Masaka_01.rtf - 21:2 [R3: Do you think you can even ..]  (70:72)   (Super)
Codes:	[FGD 2.1 Signs - Family: FGD 2.0 Signs of SA] 
No memos

R3: Do you think you can even tell where it has gone? You will only be aware that through the signs you see from the child, you will be disturbed by the way he/she looks.
R4: The eyes turn pale, his/her skin becomes elastic.
R5: That elasticity of the skins changes; meaning that the child has no water

P21: FGD_Severe_Anaemia_Masaka_01.rtf - 21:3 [R5: When you press the child w..]  (74:76)   (Super)
Codes:	[FGD 2.1 Signs - Family: FGD 2.0 Signs of SA] 
No memos

R5: When you press the child with your finger, his/her body creates a pitting edema (omubiri gukamu).
F: So it creates a pitting edema
All Rs: Yes it does

P21: FGD_Severe_Anaemia_Masaka_01.rtf - 21:4 [R3: their feet also swell R4: ..]  (83:88)   (Super)
Codes:	[FGD 2.1 Signs - Family: FGD 2.0 Signs of SA] 
No memos

R3: their feet also swell
R4: Together with the belly / abdomen
F: The abdomen swells but this is not because they are very much satisfied
R5: It is usually the feet of the pregnant women that get swollen
R2: No, even the children's feet get swollen as long as they have got a severe anemia. When you touch them, you fill a depression in their bodies.
R3: Some even look in the palms to tell whether the child has severe anemia

P21: FGD_Severe_Anaemia_Masaka_01.rtf - 21:5 [F: What I'm trying to say is t..]  (95:97)   (Super)
Codes:	[FGD 2.1 Signs - Family: FGD 2.0 Signs of SA] 
No memos

F: What I'm trying to say is that you don't need to go to class to be taught that the signs of severe anemia among children.
R4: The reason is that you can easily see it
R2: That's true, whatever the child goes through when sick of severe anemia gives you a lesson to learn. Whenever the mother/care taker takes the child to the hospital, she gets more conversant with the signs of the disease.

P22: FGD_Severe_Anaemia_Masaka_02.rtf - 22:4 [F: Now, you as mothers; what a..]  (75:80)   (Super)
Codes:	[FGD 2.1 Signs - Family: FGD 2.0 Signs of SA] 
No memos

F: Now, you as mothers; what are the signs of a child with severe anemia? Everyone will tell me what she sees. Oh I have forgotten your name, ……..yes Mariam.
R1; she turns to yellow and the eyes becomes pale.
F: The eyes becomes pale?
R4: Yes.
F: Another one.
R2: He becomes swollen.

P22: FGD_Severe_Anaemia_Masaka_02.rtf - 22:5 [F: When you say that she becom..]  (81:91)   (Super)
Codes:	[FGD 2.1 Signs - Family: FGD 2.0 Signs of SA] 
No memos

F: When you say that she becomes yellow, what becomes yellow?
R1: the whole body, he may be dark, but the color turns to yellow and with very pale (white) eyes.
F: What else do you see?
R7: He becomes swollen
F: She becomes swollen?
R7: Yes
R6; the whole body changes color.
F: Apart from changing color (kuwolubara) what else?
R1: She changes the color.
F: Already mentioned.
R: The feet became swollen.

P22: FGD_Severe_Anaemia_Masaka_02.rtf - 22:6 [How do the eyes look like? R4:..]  (94:104)   (Super)
Codes:	[FGD 2.1 Signs - Family: FGD 2.0 Signs of SA] 
No memos

How do the eyes look like?
R4: The downer part of the eye that is always red changes to white (pale).
F: What else?
R5: Even for the Aunt we used the eyes.
F: How were they looking like?
R6: pale.
F: pale like what?
R6: Where there is red in the eye,…..becomes white (pale).
(..Pause...)
F: Where does it go?
R6: It disappears.

FGD 2.2 Symptoms
______________________________________________________________________

P17: FGD_Severe_Anaemia_Hoima_01.rtf - 17:6 [SA; How about when he is old? ..]  (43:44)   (Super)
Codes:	[FGD 2.2 Symptoms - Family: FGD 2.0 Signs of SA] 
No memos

SA; How about when he is old?
R4; Mine tells me that he sees the world rotating. Meaning dizziness.  And he likes sunlight or if it's not he remains in the kitchen worming on fire.

P18: FGD_Severe_Anaemia_Hoima_02.rtf - 18:5 [R3; I wish to add on….mmm… som..]  (51:51)   (Super)
Codes:	[FGD 2.2 Symptoms - Family: FGD 2.0 Signs of SA] 
No memos

R3; I wish to add on….mmm… sometimes she refuses food and drinks but continue sleeping time after time…mmm….and is weak. Sometimes she urinates yellow urine..mmm….


Code Family: FGD 4.0 Causes of SA
______________________________________________________________________

HU:	Local Perceptions of Childhood Severe Anaemia_FA_January 2018
File:	 [C:\Users\Florence\Desktop\Local Perceptions of Childhood Severe Anaemia_FA_January 2018.hpr7]
Edited by:	Super
Date/Time:	2018-01-05 09:58:09
______________________________________________________________________

Created: 2017-12-18 09:43:37 (Super) 
Codes (6):	[FGD 4.1 Causes of SA in children] [FGD 4.2 Blood sucking agents] [FGD 4.3 Evil spirits] [FGD 4.4 Enlarged spleen] [FGD 4.5 Processes leading to SA] [FGD 4.6 Causes of SA_ranked]
Quotation(s): 81
______________________________________________________________________

FGD 4.1 Causes of SA in children
______________________________________________________________________

P17: FGD_Severe_Anaemia_Hoima_01.rtf - 17:8 [R2; sometimes a child gets sic..]  (49:49)   (Super)
Codes:	[FGD 4.1 Causes of SA in children - Family: FGD 4.0 Causes of SA] 
No memos

R2; sometimes a child gets sickness lets diarrhea or malaria and the parents don't go to seek for treatment. They remain with them at home without taking them to the hospital. Mm.. so that child spends long time with sickness and loses blood. And when you reach the hospital they ask you whether you have been seeking for medical care…mmm..

P17: FGD_Severe_Anaemia_Hoima_01.rtf - 17:9 [R1; I think poor feeding cause..]  (50:50)   (Super)
Codes:	[FGD 4.1 Causes of SA in children - Family: FGD 4.0 Causes of SA] 
No memos

R1; I think poor feeding causes severe anemia. Sometimes you find that we give a child one type of food. Everytime cassava, everytime sweet potatoes and don't put greens, yet it restores blood. Not even boil beans. Every time you give fried food yet when you fry you finish all the food nutrients…mm…ee.

P17: FGD_Severe_Anaemia_Hoima_01.rtf - 17:10 [SA; Any other thought? R4; wha..]  (52:53)   (Super)
Codes:	[FGD 4.1 Causes of SA in children - Family: FGD 4.0 Causes of SA] 
No memos

SA; Any other thought?
R4; what makes children lose blood…mmm…there are some parents who are irresponsible. (…loughs…) but me am responsible...mm… the child becomes sick and she goes to a health center and they say no medicine go and buy. He pases by a clinic and buys like panadol she give a child medicine and neither feeds him well nor gives him good drinks. So from there a child worsens and reaching the hospital again the child has no blood.

P18: FGD_Severe_Anaemia_Hoima_02.rtf - 18:10 [Res; Like this..mmm.. that's w..]  (69:69)   (Super)
Codes:	[FGD 4.1 Causes of SA in children - Family: FGD 4.0 Causes of SA] 
No memos

Res; Like this..mmm.. that's what I said that sometimes we are not informed because like dodo doesn't need having money to buy it . But because we parents have little understanding we do not secure time to provide proper food for these children. This is also one of the causes of severe anemia because the child has been weak and the body cells are not strong enough to defend.

P18: FGD_Severe_Anaemia_Hoima_02.rtf - 18:11 [SA; ok, what are the other cau..]  (78:79)   (Super)
Codes:	[FGD 4.1 Causes of SA in children - Family: FGD 4.0 Causes of SA] 
No memos

SA; ok, what are the other causes of severe anemia. We have already seen, malaria, poor feeding, what else you think?
Res; Poor care.

P19: FGD_Severe_Anaemia_Jinja_01.rtf - 19:10 [in your opinion, according to ..]  (79:82)   (Super)
Codes:	[FGD 4.1 Causes of SA in children - Family: FGD 4.0 Causes of SA] 
No memos

in your opinion, according to what we have heard or experienced, what do we believe to be the cause of anemia in children? What are the causes of childhood severe anemia? Hajira…
Respondent: some times its poor feeding…
Moderator: poor feeding, how?
Respondent: Like you have that child and they get slight malaria and you take to the hospital. You are administering strong tablets but the feeding is not good…….

P19: FGD_Severe_Anaemia_Jinja_01.rtf - 19:12 [Moderator: Now lets go back, a..]  (105:107)   (Super)
Codes:	[FGD 4.1 Causes of SA in children - Family: FGD 4.0 Causes of SA] 
No memos

Moderator: Now lets go back, apart from poor feeding, what is the other reason that causes severe anemia in children……yes, Annet B….
Respondent: Another reason is that us parents, we may be negligent with our children…feeding is not proper, the treatment is not good and the child ends up getting severe anemia……
Moderator: You have talked about poor feeding again!…..now elaborate on the treatment that you have mentioned ……

P19: FGD_Severe_Anaemia_Jinja_01.rtf - 19:13 [Respondent: Me I think that ev..]  (110:112)   (Super)
Codes:	[FGD 4.1 Causes of SA in children - Family: FGD 4.0 Causes of SA] 
No memos

Respondent: Me I think that even malaria….the child might get malaria and when you take them to hospital, they tell you he has severe anemia….
Moderator: but the cause is because of malaria............?
Respondent: mmmmm…yes

P20: FGD_Severe_Anaemia_Jinja_02.rtf - 20:7 [Respondents: the child may not..]  (82:88)   (Super)
Codes:	[FGD 4.1 Causes of SA in children - Family: FGD 4.0 Causes of SA] [FGD 4.5 Processes leading to SA - Family: FGD 4.0 Causes of SA] 
No memos

Respondents: the child may not be feeding well....the child feeds poorly..........
Moderator: How?.....
Respondent: i am still explaining.....kale…..like the child could be eating leftover food and most when it too cold,....no tea to warm the child.... not eating a balanced diet as well, like eating well......and in the end the child becomes malnourished......
Moderator: what did you say?.......
Respondent: eating sand.......
Moderators: Not eating well!! …what do you mean? okay....lets first explore the issue of poor feeding.......when you say that poor feeding, what do you mean?....
Respondents: I think the poor feeding she is talking about is eating posho lunch, supper, lunch, supper ......no....no...no that is not bad feeding.....i explained and said that the child could be feeding on left over foods every morning without tea......

P20: FGD_Severe_Anaemia_Jinja_02.rtf - 20:8 [Respondents (in chorus) musawo..]  (90:92)   (Super)
Codes:	[FGD 4.1 Causes of SA in children - Family: FGD 4.0 Causes of SA] 
No memos

Respondents (in chorus) musawo eating dirty things...not eating a balanced diet also....like feeding a child on body building and energy giving foods....those that the child can eat and the body builds the blood.....so that the child does not suffer from severe anemia...
Moderator: what are those foods that build blood?
Respondents: (in chorus) like eating eggs, bean soup, greens, fish, milk, silver fish, posho, ground nuts, cassava, avocado, unroasted pounded ground nuts and all others make up a balanced diet and build blood.......... Another thing that can make a child have severe anemia is having malaria all the time where every time the child's temperature is high something like taht

P20: FGD_Severe_Anaemia_Jinja_02.rtf - 20:9 [Respondents: the one caused by..]  (94:96)   (Super)
Codes:	[FGD 4.1 Causes of SA in children - Family: FGD 4.0 Causes of SA] 
No memos

Respondents: the one caused by mosquitoes whereby when they are sick they don't eat and the end result is Severe anemia.
Moderator: so you said when the child gets malaria, what .....
Respondent: it may cause severe anemia because earlier on we said that the child's temperature may go up and if they get that malaria often the child will not be eating, not happy and lively..and in the end the child loses blood...

P20: FGD_Severe_Anaemia_Jinja_02.rtf - 20:10 [Moderator: How does it cause s..]  (102:103)   (Super)
Codes:	[FGD 4.1 Causes of SA in children - Family: FGD 4.0 Causes of SA] 
No memos

Moderator: How does it cause severe anemia
Respondent: the child might be weak all the time and produce mucus that is green which can cause severe anemia......what is wrong with this child who is ever having flu?..........and in the end that child will waste away..... 

P20: FGD_Severe_Anaemia_Jinja_02.rtf - 20:11 [Respondents: to begin with mal..]  (109:109)   (Super)
Codes:	[FGD 4.1 Causes of SA in children - Family: FGD 4.0 Causes of SA] [FGD 4.5 Processes leading to SA - Family: FGD 4.0 Causes of SA] 
No memos

Respondents: to begin with malaria, i say that when you take this child for example like today and you take to hospital and the child is treated, in most cases when given treatment, and we try the first time and the child feels somehow better, if we stop giving drugs and after one week they again fall sick but remember when the child is sick, he or she does not eat so if they are to eat they eat little. This makes the blood building low because the child is sick and is not eating well

P20: FGD_Severe_Anaemia_Jinja_02.rtf - 20:22 [Moderator: at the start, someo..]  (189:194)   (Super)
Codes:	[FGD 4.1 Causes of SA in children - Family: FGD 4.0 Causes of SA] 
No memos

Moderator: at the start, someone talked about enkaaka (jaundice)….how does that cause a child to have  severe anemia?
Respondents: Enkaaka (meaning Jaundice) is yellow fever in English, can first talk about how you tell that the child has got severe anemai?
Moderator: it's okay, you can begin with anything….
Respondents: Okay let me first begin with how you can tell…… first and foremost when the child is passing out yellow urine, eyes turn yellow and the child does not eat like you people who studied when a someone its food there is process that food goes through for one to get blood.
Moderator: does that make a child get severe anemia?
Respondents: yes ……because the child is not eating..

P21: FGD_Severe_Anaemia_Masaka_01.rtf - 21:8 [4.0 In our next section, I wou..]  (140:143)   (Super)
Codes:	[FGD 4.1 Causes of SA in children - Family: FGD 4.0 Causes of SA] 
No memos

4.0 In our next section, I would like you to share about what you believe causes severe anemia in children
F: What do you think cause severe anemia in children?
R1: I think it could be poor feeding/nutrition
R4: and also the failure to take the child for hospital treatment i.e giving home remedies rather than take him or her to the hospital

P21: FGD_Severe_Anaemia_Masaka_01.rtf - 21:9 [4: Worms also cause severe ane..]  (146:147)   (Super)
Codes:	[FGD 4.1 Causes of SA in children - Family: FGD 4.0 Causes of SA] 
No memos

4: Worms also cause severe anemia.
R1: But the most common cause is poor feeding and the failure to get treatment in time 

P21: FGD_Severe_Anaemia_Masaka_01.rtf - 21:12 [R4: You take the medicine and ..]  (164:164)   (Super)
Codes:	[FGD 4.1 Causes of SA in children - Family: FGD 4.0 Causes of SA] [FGD 4.5 Processes leading to SA - Family: FGD 4.0 Causes of SA] 
No memos

R4: You take the medicine and give to the child, however, the child would have been poorly treated, and the malaria then remains in the body and affects him/her. So the more you poorly treat the malaria, the more it sucks blood from the child and you will see the child's health situation worsening steadily. I have ever had a sick child and I took her for treatment in these small clinics but the child's health situation worsened steadily and yet the nurse could not tell me whether she will manage to treat the child's condition or not. She told me that we should put the child on a drip which she was also not aware of whether it will be helpful in treating my child or not until when my fellow women referred me to Masaka referral hospital. I went back to this nurse and told her to write for me a note indicating the medicine that she has been giving to my child so that I can take my child to Masaka. On reaching the hospital, they discovered that the child had a severe anemia and yet this Nurse had told me nothing relating to this; but just went on with her treatment.

P21: FGD_Severe_Anaemia_Masaka_01.rtf - 21:14 [F: Can you tell me more about ..]  (172:173)   (Super)
Codes:	[FGD 4.1 Causes of SA in children - Family: FGD 4.0 Causes of SA] 
No memos

F: Can you tell me more about that? How does malaria brings about the severe anemia?
R3: it depends on the kind of fever, there is one that leads to convulsions 

P21: FGD_Severe_Anaemia_Masaka_01.rtf - 21:15 [F: which kind of fever leads t..]  (178:179)   (Super)
Codes:	[FGD 4.1 Causes of SA in children - Family: FGD 4.0 Causes of SA] 
No memos

F: which kind of fever leads to severe anemia?
R5: I think it's mainly typhoid.

P21: FGD_Severe_Anaemia_Masaka_01.rtf - 21:16 [R4: The failure to finish the ..]  (187:187)   (Super)
Codes:	[FGD 4.1 Causes of SA in children - Family: FGD 4.0 Causes of SA] [FGD 4.5 Processes leading to SA - Family: FGD 4.0 Causes of SA] 
No memos

R4: The failure to finish the dose is what we consider as the poorly treated malaria. You might choose to buy medicine from a drug-shop, start the dose but then fail to finish it. Children must be given 6 tabs of Coartem but one may first give the child 3 tabs but when they see that there is no change in the child's condition, they will change the child's treatment and when it also fails out they again change treatment instead of finishing the first dose of Coartem which they had earlier started hence leading to poorly treated malaria.

P21: FGD_Severe_Anaemia_Masaka_01.rtf - 21:18 [F: Let us go back a bit; you s..]  (219:226)   (Super)
Codes:	[FGD 4.1 Causes of SA in children - Family: FGD 4.0 Causes of SA] 
No memos

F: Let us go back a bit; you said that one of the causes of severe anemia is poor feeding. What do you mean by poor feeding?
R3: Giving the child cold food (amawolu)
R5: You give them food which is cold very early in the morning 
R6: What about drinking tea without sugar?
R5: Poor feeding is all about failure to give the child a balanced diet 
R6: Children starting their day with eating the cold food (ggolu), they are given this food even before they can wash their face.
R4: If you cook for the child food without salt in it, there is no silver fish or soup in it then I will regard that as poor feeding, the child's health cannot improve even when it means feeding them for the entire month on that diet.
R5: Aren't we told that we have to change the child' diet each day; take for example giving them millet porridge with milk. Haven't you ever taken the child off breast milk and they become stunted?

P22: FGD_Severe_Anaemia_Masaka_02.rtf - 22:7 [F: Let's take another step; wh..]  (119:122)   (Super)
Codes:	[FGD 4.1 Causes of SA in children - Family: FGD 4.0 Causes of SA] 
No memos

F: Let's take another step; what causes severe anemia among children?
R3: Poor feeding.
F: What about poor feeding?
R3: When the child is not feeding on the diet that builds blood. To find that the food that he eats does not suit the requirements of the body.

P22: FGD_Severe_Anaemia_Masaka_02.rtf - 22:11 [: Say what you know or what yo..]  (138:140)   (Super)
Codes:	[FGD 4.1 Causes of SA in children - Family: FGD 4.0 Causes of SA] [FGD 4.5 Processes leading to SA - Family: FGD 4.0 Causes of SA] 
No memos

: Say what you know or what you hear.
R5; Even poverty causes SA.
R1; poor health care. The child may suffer from malaria and the parent doesn't care and says aaa that's how he is always. So the malaria becomes severe.

P22: FGD_Severe_Anaemia_Masaka_02.rtf - 22:32 [R7: even measles can cause sev..]  (374:374)   (Super)
Codes:	[FGD 4.1 Causes of SA in children - Family: FGD 4.0 Causes of SA] 
No memos

R7: even measles can cause severe anemia. 

FGD 4.2 Blood sucking agents
______________________________________________________________________

P18: FGD_Severe_Anaemia_Hoima_02.rtf - 18:13 [A; What else that you thin suc..]  (89:95)   (Super)
Codes:	[FGD 4.2 Blood sucking agents - Family: FGD 4.0 Causes of SA] 
No memos

A; What else that you thin sucks blood?
Res; There is another thing that keeps pulling him. When he has just slept, it keeps pulling him. He may be with little malaria but thing continues pulling him….(…….referring to seizures)
SA; which thing is that?
Res; (…All laughs…) when you take him to the hospital they tell you it's malaria.
SA; for you what do you think it is?
Res; for me I have met some other people who say this child has been bewitched. And they keep using herbals at the end of it all they still find that the child has no blood he remains there when they are removing those traditional things but at the end he has no blood.
SA; what do you call those things that suck blood

P18: FGD_Severe_Anaemia_Hoima_02.rtf - 18:14 [Res; they are witch-craft. The..]  (96:100)   (Super)
Codes:	[FGD 4.2 Blood sucking agents - Family: FGD 4.0 Causes of SA] 
No memos

Res; they are witch-craft. They are called “ebihara” in runyoro and you find that when you insist and take the child to the hospital and they inject him, he dies.
SA; they are called ebihara?
All; yes; they are always in the stomach (Abdomen)
SA; and they do what in the abdomen?
All; they suck blood.

P18: FGD_Severe_Anaemia_Hoima_02.rtf - 18:15 [SA; Try to explain more about ..]  (102:103)   (Super)
Codes:	[FGD 4.2 Blood sucking agents - Family: FGD 4.0 Causes of SA] 
No memos

SA; Try to explain more about “ebihara”. What are they? …..Are they spirits?
Res; It is witch-craft. The abdomen swells, the child neither drinks nor eats but keeps asleep so they suck blood.

P18: FGD_Severe_Anaemia_Hoima_02.rtf - 18:16 [Res; they also say false teeth..]  (104:108)   (Super)
Codes:	[FGD 4.2 Blood sucking agents - Family: FGD 4.0 Causes of SA] 
No memos

Res; they also say false teeth (ebiino) suck blood and when you don't remove them the child dies.
SA; What?
Res; These “false teeth” that they remove from children. 
SA; how they take away the blood?
Res; they make a child turn yellow. I took the child to the hospital; it didn't work out and I took them (to a herbalist) to be removed. They are also in the category of witch-craft. They may be in kigorobya “this is one of the villages in this place” but if you go to kitoba village they may not be there. So they are also witch craft. They say they bewitch them to a pregnant woman, and when she produces they attack / affect the child.

P18: FGD_Severe_Anaemia_Hoima_02.rtf - 18:17 [SA; is there any other thing t..]  (116:117)   (Super)
Codes:	[FGD 4.2 Blood sucking agents - Family: FGD 4.0 Causes of SA] [FGD 4.5 Processes leading to SA - Family: FGD 4.0 Causes of SA] 
No memos

SA; is there any other thing that sucks blood?
Even when the child is developing teeth...mmm... he first experiences diarrhea for some time and in that process when you take a child to the hospital they tell you the child has no enough blood

P19: FGD_Severe_Anaemia_Jinja_01.rtf - 19:18 [Respondents: that one could be..]  (167:167)   (Super)
Codes:	[FGD 4.2 Blood sucking agents - Family: FGD 4.0 Causes of SA] [FGD 4.3 Evil spirits - Family: FGD 4.0 Causes of SA] 
No memos

Respondents: that one could be having clan spirits……ahaa (no, says another respondent....)…nothing sucks blood from him……but that child……

P19: FGD_Severe_Anaemia_Jinja_01.rtf - 19:21 [Respondents (all) Majority of ..]  (177:177)   (Super)
Codes:	[FGD 4.2 Blood sucking agents - Family: FGD 4.0 Causes of SA] 
No memos

Respondents (all) Majority of them (spirits) are just sent. Those that suck blood….there those who buy wealth…ahaaa….…I have desired to have riches…..

P19: FGD_Severe_Anaemia_Jinja_01.rtf - 19:22 [Moderator: You said when these..]  (182:189)   (Super)
Codes:	[FGD 4.2 Blood sucking agents - Family: FGD 4.0 Causes of SA] [FGD 4.3 Evil spirits - Family: FGD 4.0 Causes of SA] 
No memos

Moderator: You said when these things come…..don't they have a name?
Respondents: (in chorus) “Majini”, “mawembe”, “bitega”……..
Moderator: wait a minute. One by one…..
Respondents: “mijini”, “bitega”, “mawembe”, “ misambwa”
Moderator: Which of these sucks blood most?
Respondent: wooooh! Hihihi……”Kitega”…..
Moderator: Kitega?........How does it suck?
Respondent: the kitega…….you can see something small….it can be like a black-anti, or a snake, or a lizard…..it bites you just a little bit and you fall there and then……..

P19: FGD_Severe_Anaemia_Jinja_01.rtf - 19:23 [Moderators: So all these four ..]  (190:197)   (Super)
Codes:	[FGD 4.2 Blood sucking agents - Family: FGD 4.0 Causes of SA] 
No memos

Moderators: So all these four categories of spirits are just sent?
Respondents: (in chorus) yiiiii (yes)…..they are just sent…….and whenever you see a thing such as; a black-anti, snake or lizard, just know / suspect there is something behind it…… (these are agents / carriers of evils pirits) ……
Moderator: who commands them (spirits)
Respondents (in chorus) anyone who wants to harm you…..
Moderator: They are just commanded to come and do what the commander wants?
Respondents (in chorus)…….. mmmm…… (yes)…….they come and do what they have been sent to do…………..
Moderator: Which work?
Respondents: (in chorus) ….sucking the blood……..

P20: FGD_Severe_Anaemia_Jinja_02.rtf - 20:15 [Moderators: aha......we are st..]  (145:150)   (Super)
Codes:	[FGD 4.2 Blood sucking agents - Family: FGD 4.0 Causes of SA] [FGD 4.3 Evil spirits - Family: FGD 4.0 Causes of SA] 
No memos

Moderators: aha......we are still discussing the causes....causes of severe anemia....are there local spirits that cause severe anemia in children?.....
Respondents: yes we have ..”Ebijini”, “amayembe”, and the “agazimuzimu (ghosts)” and even “illuminati” also sucks blood...hehehe...that they have sucked blood..... 
Moderator: what? emijini.....what else? you said emijini, mawembe......
Respondent: agazimuzimu (ghosts) hmihmi......
Moderator: don't they have a name?
Respondents: Nga (such as) “lubaale”...hahahaha.........

P20: FGD_Severe_Anaemia_Jinja_02.rtf - 20:16 [Moderator: Do we know of any p..]  (151:152)   (Super)
Codes:	[FGD 4.2 Blood sucking agents - Family: FGD 4.0 Causes of SA] 
No memos

Moderator: Do we know of any particular evil spirit that sucks blood out of children?
Respondent: anti she asked for the name....the mawembe....”amawembe” is the most common...agazimu (ghosts).... there those things that have just come these days...alminati...mbu they suck blood from a person......

P20: FGD_Severe_Anaemia_Jinja_02.rtf - 20:21 [Moderator: apart from the abov..]  (185:186)   (Super)
Codes:	[FGD 4.2 Blood sucking agents - Family: FGD 4.0 Causes of SA] 
No memos

Moderator: apart from the above you have mentioned of Amayembe, the ghosts, ancestral spirits, illuminati, what else do think sucks blood from the child?
Respondents: Hmhmmmmmm..kubuguyala, .. when you live the child in the cold, as in not covering them at night, they feel cold and they start shivering. What follows is they fall sick and get severe anemia. Even drinking alcohol…….hmmmm.

P21: FGD_Severe_Anaemia_Masaka_01.rtf - 21:17 [F: How does sickle cell lead t..]  (200:202)   (Super)
Codes:	[FGD 4.2 Blood sucking agents - Family: FGD 4.0 Causes of SA] [FGD 4.5 Processes leading to SA - Family: FGD 4.0 Causes of SA] 
No memos

F: How does sickle cell lead to severe anemia? What is sickle cells?
R5: One of my fellow respondents explained sickle cells as a recurrent disease to imply that it doesn't heal but continuously leads to severe anemia.
R2: it is like parasite feeding on someone daily.

P21: FGD_Severe_Anaemia_Masaka_01.rtf - 21:21 [R2: It drinks (or sucks) a lot..]  (232:232)   (Super)
Codes:	[FGD 4.2 Blood sucking agents - Family: FGD 4.0 Causes of SA] 
No memos

R2: It drinks (or sucks) a lot of blood

P21: FGD_Severe_Anaemia_Masaka_01.rtf - 21:22 [F: How does an enlarged spleen..]  (235:236)   (Super)
Codes:	[FGD 4.2 Blood sucking agents - Family: FGD 4.0 Causes of SA] [FGD 4.5 Processes leading to SA - Family: FGD 4.0 Causes of SA] 
No memos

F: How does an enlarged spleen suck blood?
R1: I hear it is positioned near the heart from where it sucks blood.

P22: FGD_Severe_Anaemia_Masaka_02.rtf - 22:14 [F: Where does it suck the bloo..]  (175:180)   (Super)
Codes:	[FGD 4.2 Blood sucking agents - Family: FGD 4.0 Causes of SA] 
No memos

F: Where does it suck the blood from?
R1: It sucks it from the whole body. The Baganda cut it and then the child stabilizes and even starts eating. But before it is cut it can't allow the child to eat properly.
F: When they cut it, what do they apply?
R2: There is an herbal they apply there.
F: Don't you know its name?
R5: Mmmm.. I don't know.

P22: FGD_Severe_Anaemia_Masaka_02.rtf - 22:16 [R3: AIDS is the worst of all, ..]  (206:206)   (Super)
Codes:	[FGD 4.2 Blood sucking agents - Family: FGD 4.0 Causes of SA] 
No memos

R3: AIDS is the worst of all, in sucking blood. There is a neighbor's child. She was born with HIV but every time she was anemic. She eats but doesn't fatten

FGD 4.3 Evil spirits
______________________________________________________________________

P17: FGD_Severe_Anaemia_Hoima_01.rtf - 17:16 [SA; What I wanted to know; the..]  (79:81)   (Super)
Codes:	[FGD 4.3 Evil spirits - Family: FGD 4.0 Causes of SA] [FGD 5.1 Health care seeking behaviour patterns for SA - Family: FGD 5.0 Care seeking for children with SA] 
No memos

SA; What I wanted to know; there are other people who say that the evil spirits and witch craft suck blood. What do you know about this?
Res; we hear that from other people. That it is Satan and then they go to prayer warriors yet the child has severe anemia…mm… or they remain at home using home remedies and finally the child dies. 
Res; when a child becomes sick everyone talks to his mind, others that the child is bewitched; take the child for prayers but for me I decide to take mine to the hospital….mmm…

P19: FGD_Severe_Anaemia_Jinja_01.rtf - 19:18 [Respondents: that one could be..]  (167:167)   (Super)
Codes:	[FGD 4.2 Blood sucking agents - Family: FGD 4.0 Causes of SA] [FGD 4.3 Evil spirits - Family: FGD 4.0 Causes of SA] 
No memos

Respondents: that one could be having clan spirits……ahaa (no, says another respondent....)…nothing sucks blood from him……but that child……

P19: FGD_Severe_Anaemia_Jinja_01.rtf - 19:19 [Respondents: mmmmh……bedbugs do..]  (173:173)   (Super)
Codes:	[FGD 4.3 Evil spirits - Family: FGD 4.0 Causes of SA] 
No memos

Respondents: mmmmh……bedbugs don't suck blood out of that child unless they have been sacrificed or they have some spirits……(all)…mmmm…..there is a child having spirits……but there is nothing that suck blood out of the child……

P19: FGD_Severe_Anaemia_Jinja_01.rtf - 19:20 [Moderators: lets explain……leav..]  (174:175)   (Super)
Codes:	[FGD 4.3 Evil spirits - Family: FGD 4.0 Causes of SA] 
No memos

Moderators: lets explain……leave alone the medical things…talk about the evil spirits……those who have “evil spirits”….what happens to them …..?
Respondents: There an instance where the evil spirits want blood ….where they command you to sacrifice….and they choose a particular child…in that case, the spirit just suck the blood out of the child and they get dry (severe anemia) and even when you put a drip of water, the child does not survive…..even when the child gets a blood transfusion, it does not go in the body……(other respondents) say it goes in but it all goes out….the transfusion that they give at the hospital….the child consumes drip after another because as they put the blood on, it (evil spirit) sucks it all out.....................

P19: FGD_Severe_Anaemia_Jinja_01.rtf - 19:22 [Moderator: You said when these..]  (182:189)   (Super)
Codes:	[FGD 4.2 Blood sucking agents - Family: FGD 4.0 Causes of SA] [FGD 4.3 Evil spirits - Family: FGD 4.0 Causes of SA] 
No memos

Moderator: You said when these things come…..don't they have a name?
Respondents: (in chorus) “Majini”, “mawembe”, “bitega”……..
Moderator: wait a minute. One by one…..
Respondents: “mijini”, “bitega”, “mawembe”, “ misambwa”
Moderator: Which of these sucks blood most?
Respondent: wooooh! Hihihi……”Kitega”…..
Moderator: Kitega?........How does it suck?
Respondent: the kitega…….you can see something small….it can be like a black-anti, or a snake, or a lizard…..it bites you just a little bit and you fall there and then……..

P19: FGD_Severe_Anaemia_Jinja_01.rtf - 19:24 [Moderator: okay…..apart from k..]  (202:209)   (Super)
Codes:	[FGD 4.3 Evil spirits - Family: FGD 4.0 Causes of SA] 
No memos

Moderator: okay…..apart from kitega, what follows it in blood sucking ability? The kitega comes first, followed by?...........”amawembe”, “amajini” and “misambwa”…what follows the kitega in sucking blood?......what you know?
Respondent: I think the “misambwa”………..
Moderator: they are the ones that suck blood most?
Respondents: (in chorus) they come second………
Moderator: followed by?
Respondents: “Amawembe”………..
Moderator: amawembe……..the mijini comes last?
Respondent: mmmmm….(yes)

P20: FGD_Severe_Anaemia_Jinja_02.rtf - 20:15 [Moderators: aha......we are st..]  (145:150)   (Super)
Codes:	[FGD 4.2 Blood sucking agents - Family: FGD 4.0 Causes of SA] [FGD 4.3 Evil spirits - Family: FGD 4.0 Causes of SA] 
No memos

Moderators: aha......we are still discussing the causes....causes of severe anemia....are there local spirits that cause severe anemia in children?.....
Respondents: yes we have ..”Ebijini”, “amayembe”, and the “agazimuzimu (ghosts)” and even “illuminati” also sucks blood...hehehe...that they have sucked blood..... 
Moderator: what? emijini.....what else? you said emijini, mawembe......
Respondent: agazimuzimu (ghosts) hmihmi......
Moderator: don't they have a name?
Respondents: Nga (such as) “lubaale”...hahahaha.........

P20: FGD_Severe_Anaemia_Jinja_02.rtf - 20:18 [Moderator: Aha, meaning they e..]  (163:166)   (Super)
Codes:	[FGD 4.3 Evil spirits - Family: FGD 4.0 Causes of SA] 
No memos

Moderator: Aha, meaning they exist, so how to they suck blood?...you had said that they come through the wind
Respondents: they come in form of wind, sometimes even when the child is taken to the hospital, they test and they don't find the cause of sickness but child keeps loosing blood which confirms that they are spirits sucking blood from that child.....if you go to the hospital, you just believe.....this one does not like me.....hahahaha.........and you just suspect when you are not even sure.....
Moderator: eeeh...you just suspect?
Respondent: (in chorus) yes....sometimes you don't even have evidence and you just suspect that i think it is this person who does not like me......anti what brings that is that you can go to the hospital and they test the child and they don't detect any disease.....they tell you this child is normal....or they get a blood transfusion but the blood goes all the time (the child gets severe anemia all the time) and you will wonder...are there things sucking blood out of him..........hahaha...... 

P20: FGD_Severe_Anaemia_Jinja_02.rtf - 20:19 [Moderator: Do they (ancestral ..]  (173:174)   (Super)
Codes:	[FGD 4.3 Evil spirits - Family: FGD 4.0 Causes of SA] [FGD 4.5 Processes leading to SA - Family: FGD 4.0 Causes of SA] 
No memos

Moderator: Do they (ancestral spirits) also suck blood?.....how do they choose the person they suck blood from? because we mentioned amawembe, emijini, emisambwa.....what are ancestral spirits?
Respondents: They also suck...those walugonos hehehee........and they can make the child not to walk and they say that child has walugono.... that it sits on him.....i don't know whether the child feels the ghost sitting on them.......and that person stays knowing that they are not alone.......

P20: FGD_Severe_Anaemia_Jinja_02.rtf - 20:20 [Moderator: so what is walugono..]  (177:182)   (Super)
Codes:	[FGD 4.3 Evil spirits - Family: FGD 4.0 Causes of SA] 
No memos

Moderator: so what is walugono?.......
Respondents: they are from the clan......those were brought by ancestors.........and so they go on following those in the clan......
Moderator: is it a ghost?.......You said spirits of ancestors follow, why do the spirits follow this and not the other person?
Respondents: because you are a member of the clan......yes they just like a person and that is the one whose blood is sucked..
Moderator: following one…….hmmmmm….could it be that he or she has committed a crime?
Respondents: No, it is just because they have liked you.....there is no reason or crime one commits.

P21: FGD_Severe_Anaemia_Masaka_01.rtf - 21:24 [F: As we continue, someone tol..]  (303:312)   (Super)
Codes:	[FGD 4.3 Evil spirits - Family: FGD 4.0 Causes of SA] 
No memos

F: As we continue, someone told us that sometimes there are evil spirits that suck blood. Do you have an idea about this?
R3: you mean the devilish things
All Rs: those are very many in this village
R4: and those ones can even kill a child in just one day.
F: What name is given to them?
R3: “Amayembe”
R5: Ghosts
R3: Can someone send you “ejjembe” and it fails to suck blood out of you? That is why even if it is an adult person who continuously loses blood after a blood transfusion is suspected to have been sent “amayembe.” They must be having something sucking their blood 
R4: The blood that is restored through the blood transfusion is what it sucks out.
R3: These evil spirits that suck blood do exist, they are so common like beans these days.

P21: FGD_Severe_Anaemia_Masaka_01.rtf - 21:25 [R3: The Baganda will have alre..]  (315:323)   (Super)
Codes:	[FGD 4.3 Evil spirits - Family: FGD 4.0 Causes of SA] 
No memos

R3: The Baganda will have already known the cause and proceed or take the next step.
F: What other step do they take?
R3: Go to the traditional healers.
F: How do you tell that it could be evil spirits that are sucking blood?
R2: you treat the child on several occasions but find that there is no improvement in the child's health.
R1: They get “stuttles”
F: what do stuttles mean?
R2: To get shocked 
R4: When a child gets shocked, as a parent it will come to your knowledge that this is not a disease/ infection as such that can be treated in hospital. 

P22: FGD_Severe_Anaemia_Masaka_02.rtf - 22:15 [F: You told me about an enlarg..]  (195:201)   (Super)
Codes:	[FGD 4.3 Evil spirits - Family: FGD 4.0 Causes of SA] 
No memos

F: You told me about an enlarged spleen, what else do you think sucks blood?
R4: Amayembe. There is a man called Wamala, he lost a nice child 
F: Do the “mayembe” also suck the blood?
R4: Mmm..They killed Cissy's grandchild. She lost blood and they said it was the “mayembe.” (laughs)
R7: The parents were Irresponsible. It was not as a result of amayembe. The child had flue and they covered her completely failed to get air, suffocated and then she died.
F: But have you ever had of any child whose blood was sucked by the amayembe?
R4: yes there are many. (….Laughs) they administer blood and later after like two days they say he has no blood. Which means it's the “amayembe” sucking it. Like the late Martin Angume they say every blood they put would be consumed and therefore it were the amayembe.

FGD 4.4 Enlarged spleen
______________________________________________________________________

P18: FGD_Severe_Anaemia_Hoima_02.rtf - 18:3 [SA; which thing is that? R4; I..]  (39:44)   (Super)
Codes:	[FGD 2.1 Signs - Family: FGD 2.0 Signs of SA] [FGD 4.4 Enlarged spleen - Family: FGD 4.0 Causes of SA] 
No memos

SA; which thing is that?
R4; I dint know it but they say its stonelike (we call it ekibare)……referring to an enlarged spleen….
SA; which side?
Res; This side (…. As some of the respondents touch their left part of abdomen to show where it swells from…) for it doesn't come outside but swells from inside.
SA; Uhh…what else?
R4; She feels stomach ache.

P21: FGD_Severe_Anaemia_Masaka_01.rtf - 21:19 [R2: I earlier talked about it ..]  (228:228)   (Super)
Codes:	[FGD 4.4 Enlarged spleen - Family: FGD 4.0 Causes of SA] 
No memos

R2: I earlier talked about it and that is what we refer to as an enlarged spleen (ekikubuuko)

P21: FGD_Severe_Anaemia_Masaka_01.rtf - 21:20 [R5: Some people refer to it as..]  (231:231)   (Super)
Codes:	[FGD 4.4 Enlarged spleen - Family: FGD 4.0 Causes of SA] 
No memos

R5: Some people refer to it as “akabengo”

P21: FGD_Severe_Anaemia_Masaka_01.rtf - 21:23 [R1: yes, I hear the “Male enla..]  (238:244)   (Super)
Codes:	[FGD 4.4 Enlarged spleen - Family: FGD 4.0 Causes of SA] [FGD 4.5 Processes leading to SA - Family: FGD 4.0 Causes of SA] 
No memos

R1: yes, I hear the “Male enlarged spleen” (“akasajja”) rests at the upper part of the abdomen while the female one (“akakazzi”) on the lower part of the abdomen.
F: What is do you mean by enlarged spleen?
R1: the doctors tell us that is a type of fever and that they have its medicine.
F: Is it found in the stomach?
All Rs: Yes
F: What does it look like?
R3: When you touch the child in the child's tummy you can feel it, it is hard but when you take the child for treatment and they cut it and put medicine, the child will be healed of it.

P22: FGD_Severe_Anaemia_Masaka_02.rtf - 22:13 [F: Apart from the malaria para..]  (167:174)   (Super)
Codes:	[FGD 4.4 Enlarged spleen - Family: FGD 4.0 Causes of SA] 
No memos

F: Apart from the malaria parasites which you say suck blood. What else do you know sucks blood from our children?
R7: May be the traditional one. (ekimbe ekiganda)
R4: There is the enlarged spleen (ekikuubuko). (a hard swelling on the side part in the abdomen). It sucks blood.
F: Where do you find an enlarged spleen?
R3: It's like here. (She torches her left part of the abdomen, displaying where it attacks)
R7: It (enlarged spleen) comes when it is like a stone especially during coldness.
F: How does it behave?
R7: It forms a stone like swelling, and makes the child restless and keeps sucking his blood.

FGD 4.5 Processes leading to SA
______________________________________________________________________

P17: FGD_Severe_Anaemia_Hoima_01.rtf - 17:11 [How does poor feeding take awa..]  (54:55)   (Super)
Codes:	[FGD 4.5 Processes leading to SA - Family: FGD 4.0 Causes of SA] 
No memos

How does poor feeding take away the blood? One of you talked about poor feeding, another one said malaria. How does the malaria take away the blood?
R4; the way malaria takes away the blood; mm.. a child becomes sick when may be the parent doesn't have money.

P17: FGD_Severe_Anaemia_Hoima_01.rtf - 17:12 [SA; so there you think what to..]  (60:61)   (Super)
Codes:	[FGD 4.5 Processes leading to SA - Family: FGD 4.0 Causes of SA] 
No memos

SA; so there you think what took away the blood? 
Res; for me I thought it's that malaria that took it away

P17: FGD_Severe_Anaemia_Hoima_01.rtf - 17:13 [Anet; when severe malaria atta..]  (66:66)   (Super)
Codes:	[FGD 4.5 Processes leading to SA - Family: FGD 4.0 Causes of SA] 
No memos

Anet; when severe malaria attacks a child, her white blood cells reduces and this causes severe anemia…mm…

P17: FGD_Severe_Anaemia_Hoima_01.rtf - 17:14 [SA; so what happens when the w..]  (67:70)   (Super)
Codes:	[FGD 4.5 Processes leading to SA - Family: FGD 4.0 Causes of SA] 
No memos

SA; so what happens when the white blood cells are affected?
R8; the child might die
SA; ee..We are talking about severe anemia.
R8; when the white blood cells reduce the child loses blood.

P18: FGD_Severe_Anaemia_Hoima_02.rtf - 18:8 [SA; how does the malaria cause..]  (60:61)   (Super)
Codes:	[FGD 4.5 Processes leading to SA - Family: FGD 4.0 Causes of SA] 
No memos

SA; how does the malaria cause severe anemia?
R3; Because of recurring in the first, second and third weeks they weaken the child. He doesn't eat nor drink. In that way the blood is lost because when a child is sick he can't feed properly as a result he will lose blood.

P18: FGD_Severe_Anaemia_Hoima_02.rtf - 18:9 [SA; tell us what you think. R4..]  (66:67)   (Super)
Codes:	[FGD 4.5 Processes leading to SA - Family: FGD 4.0 Causes of SA] 
No memos

SA; tell us what you think.
R4; now since the child is always weak…mmm…white blood cells also become weak and can no longer have any defensive mechanism. And sometimes you find that the child become sick and we are poor even if you want to feed him well you are unable

P18: FGD_Severe_Anaemia_Hoima_02.rtf - 18:12 [SA; some people where we have ..]  (80:86)   (Super)
Codes:	[FGD 4.5 Processes leading to SA - Family: FGD 4.0 Causes of SA] 
No memos

SA; some people where we have been have been telling us there is something that sucks blood out children. Do you know anything about this?
Res;  Its malaria that sucks blood.
SA; How?
Res; because if a child becomes sick and you don't treat quickly...mmm… it brings loss of blood.
SA; How does it get lost?
Res; (…Loughs…) according to our thinking those parasites are the ones that suck blood…mmm…. Ok.
Res; for me I think that the drugs we use do not deal with the parasites, you give drugs but the malaria persists...uhhh…

P18: FGD_Severe_Anaemia_Hoima_02.rtf - 18:17 [SA; is there any other thing t..]  (116:117)   (Super)
Codes:	[FGD 4.2 Blood sucking agents - Family: FGD 4.0 Causes of SA] [FGD 4.5 Processes leading to SA - Family: FGD 4.0 Causes of SA] 
No memos

SA; is there any other thing that sucks blood?
Even when the child is developing teeth...mmm... he first experiences diarrhea for some time and in that process when you take a child to the hospital they tell you the child has no enough blood

P19: FGD_Severe_Anaemia_Jinja_01.rtf - 19:11 [Moderator: what is that feedin..]  (85:86)   (Super)
Codes:	[FGD 4.5 Processes leading to SA - Family: FGD 4.0 Causes of SA] 
No memos

Moderator: what is that feeding that is not good?
Respondent: that feeding is bad. That child should take some passion juice, ORS, Lucozade, so that that child regains quickly to bring back the life that was going. so it causes that child who has been having malaria to result into another disease that may make him be admitted and instead of a drip, they give him what…blood…..

P19: FGD_Severe_Anaemia_Jinja_01.rtf - 19:14 [Moderators: How does malaria c..]  (113:116)   (Super)
Codes:	[FGD 4.5 Processes leading to SA - Family: FGD 4.0 Causes of SA] 
No memos

Moderators: How does malaria cause severe anemia?......how does it cause severe anemia..the process….
Respondents: now the child may be home and they get sick. When they get sick you take him to hospital remember it was malaria but when you reach they tell you that he also has severe anemia…….
Moderator: so the question is, how does malaria cause severe anemia in a child?
Respondents: when you delay treatment….when you have not treated malaria, it causes a child to get severe anemia…because he becomes hot all the time..when you delay to give treatment…

P19: FGD_Severe_Anaemia_Jinja_01.rtf - 19:15 [Respondents: mhmhmhm………………when..]  (128:128)   (Super)
Codes:	[FGD 4.5 Processes leading to SA - Family: FGD 4.0 Causes of SA] 
No memos

Respondents: mhmhmhm………………when malaria finds when the body is weak, it becomes easier for the child to get severe anemia. Sometimes it happens that when a child gets malaria, they urinate bloody urine….which makes the child get severe anemia….remember….

P19: FGD_Severe_Anaemia_Jinja_01.rtf - 19:16 [Respondents: mhmhn….that is ho..]  (136:136)   (Super)
Codes:	[FGD 4.5 Processes leading to SA - Family: FGD 4.0 Causes of SA] 
No memos

Respondents: mhmhn….that is how we think…..in our thinking….and remember when a child has got malaria…in my thinking because many children when they get sick, they don't want to eat and drink….so I think that it causes severe anemia…in my thinking…

P19: FGD_Severe_Anaemia_Jinja_01.rtf - 19:17 [Respondents: Me I think that t..]  (140:140)   (Super)
Codes:	[FGD 4.5 Processes leading to SA - Family: FGD 4.0 Causes of SA] 
No memos

Respondents: Me I think that the malaria comes when it is strong (or severe) and causes severe anemia…..If finds when the body is weak (mmmmm) but when it (malaria) is severe or strong also….(mmm)…my body mat not be able to fight that disease……

P19: FGD_Severe_Anaemia_Jinja_01.rtf - 19:25 [Moderator: But do they (evil s..]  (210:215)   (Super)
Codes:	[FGD 4.5 Processes leading to SA - Family: FGD 4.0 Causes of SA] 
No memos

Moderator: But do they (evil spirits) suck blood?
Respondents (in chorus) yes……………..
Moderator: Is it (blood) their food? Or they suck and take it to the person who has sent them?
Respondent: it is like this. You don't see the blood but you just see someone die….
Moderator: my question is, on the whole body, where does it exactly suck the blood from…….?
Respondent: You might feel cold at once and your situation changes, you don't understand it and you just die……and you just see your life being drained….

P20: FGD_Severe_Anaemia_Jinja_02.rtf - 20:7 [Respondents: the child may not..]  (82:88)   (Super)
Codes:	[FGD 4.1 Causes of SA in children - Family: FGD 4.0 Causes of SA] [FGD 4.5 Processes leading to SA - Family: FGD 4.0 Causes of SA] 
No memos

Respondents: the child may not be feeding well....the child feeds poorly..........
Moderator: How?.....
Respondent: i am still explaining.....kale…..like the child could be eating leftover food and most when it too cold,....no tea to warm the child.... not eating a balanced diet as well, like eating well......and in the end the child becomes malnourished......
Moderator: what did you say?.......
Respondent: eating sand.......
Moderators: Not eating well!! …what do you mean? okay....lets first explore the issue of poor feeding.......when you say that poor feeding, what do you mean?....
Respondents: I think the poor feeding she is talking about is eating posho lunch, supper, lunch, supper ......no....no...no that is not bad feeding.....i explained and said that the child could be feeding on left over foods every morning without tea......

P20: FGD_Severe_Anaemia_Jinja_02.rtf - 20:11 [Respondents: to begin with mal..]  (109:109)   (Super)
Codes:	[FGD 4.1 Causes of SA in children - Family: FGD 4.0 Causes of SA] [FGD 4.5 Processes leading to SA - Family: FGD 4.0 Causes of SA] 
No memos

Respondents: to begin with malaria, i say that when you take this child for example like today and you take to hospital and the child is treated, in most cases when given treatment, and we try the first time and the child feels somehow better, if we stop giving drugs and after one week they again fall sick but remember when the child is sick, he or she does not eat so if they are to eat they eat little. This makes the blood building low because the child is sick and is not eating well

P20: FGD_Severe_Anaemia_Jinja_02.rtf - 20:12 [Respondents: Ok musawo this is..]  (119:119)   (Super)
Codes:	[FGD 4.5 Processes leading to SA - Family: FGD 4.0 Causes of SA] 
No memos

Respondents: Ok musawo this is what i think.......there are some children whose blood vessels are blocked due to convulsions when they fall sick ....so when the blood vessels get blocked and there is no blood circulating....it causes severe anemia......

P20: FGD_Severe_Anaemia_Jinja_02.rtf - 20:13 [Moderator: when blood vessels ..]  (122:127)   (Super)
Codes:	[FGD 4.5 Processes leading to SA - Family: FGD 4.0 Causes of SA] 
No memos

Moderator: when blood vessels are blocked, how does it cause severe anemia?
Respondents: (in chorus) Now musawo...when the child convulses and you take him or her to hospital and he is given treatment
Moderator: no don't divert...the child is convulsing......the blood vessels are contracting...what next?
Respondent: mmmm.....the vessels are blocked, it means that the flow of blood to other parts of the body reduces making them become anemic.....
Moderator: How?.
Respondent: because of the low blood circulation.........and because the blood is not circulating in other parts of the body...mmmm and in the end that the child has severe anemia......

P20: FGD_Severe_Anaemia_Jinja_02.rtf - 20:14 [Moderators: i have understood...]  (128:129)   (Super)
Codes:	[FGD 4.5 Processes leading to SA - Family: FGD 4.0 Causes of SA] 
No memos

Moderators: i have understood.....how about poor feeding, how does it cause severe anemia in a child?
Respondents: let me give an example if i earn well are well and can afford meat on daily basis, and my children  will eat only meat from time to time while the neighbour's children  will feed on greens, bean and the village child will be healthier and you will notice that this other child is all the time falling sick.......and when you take to hospital they will say the child does not have blood.......but because the child is eating meat alone which is not helping him....so diet also can cause severe anemia....it is good to give the child some beans like now it is the season.....

P20: FGD_Severe_Anaemia_Jinja_02.rtf - 20:17 [Moderator: how do mawembe caus..]  (155:158)   (Super)
Codes:	[FGD 4.5 Processes leading to SA - Family: FGD 4.0 Causes of SA] 
No memos

Moderator: how do mawembe cause severe anemia?
Respondents: ..it could be that someone doesn't like you, then they  send the spirits to come and suck blood
Moderator: If they send them....are they just commanded?...how do the spirits suck blood? do they attack the hand? where do they attack?.....do they come with a straw?
Respondents: if you have a belief that someone has sent you amawembe, even when you fall sick I will be growing thin instead of going to the hospital because I will assume that someone that sent me the spirits is the one against me even when I am sick.....mmmhm...the spirits sucking blood from me.....and as i grow thin, i am believing that this person is the one against me and they have sent mawembe to suck blood......

P20: FGD_Severe_Anaemia_Jinja_02.rtf - 20:19 [Moderator: Do they (ancestral ..]  (173:174)   (Super)
Codes:	[FGD 4.3 Evil spirits - Family: FGD 4.0 Causes of SA] [FGD 4.5 Processes leading to SA - Family: FGD 4.0 Causes of SA] 
No memos

Moderator: Do they (ancestral spirits) also suck blood?.....how do they choose the person they suck blood from? because we mentioned amawembe, emijini, emisambwa.....what are ancestral spirits?
Respondents: They also suck...those walugonos hehehee........and they can make the child not to walk and they say that child has walugono.... that it sits on him.....i don't know whether the child feels the ghost sitting on them.......and that person stays knowing that they are not alone.......

P21: FGD_Severe_Anaemia_Masaka_01.rtf - 21:10 [F: Can you talk more about the..]  (154:155)   (Super)
Codes:	[FGD 4.5 Processes leading to SA - Family: FGD 4.0 Causes of SA] 
No memos

F: Can you talk more about the diseases which you mentioned that if not treated early will lead to severe anemia?
R3: when you keep the child home while sick you need to know it as a parent that you are not a doctor and so this child might die in your hands. The child might have severe anemia without your knowledge and even if you get to know it you will not cook local herbs and give to the child and expect that they will be well. You still have to take them to the hospital to get the blood transfusion treatment

P21: FGD_Severe_Anaemia_Masaka_01.rtf - 21:12 [R4: You take the medicine and ..]  (164:164)   (Super)
Codes:	[FGD 4.1 Causes of SA in children - Family: FGD 4.0 Causes of SA] [FGD 4.5 Processes leading to SA - Family: FGD 4.0 Causes of SA] 
No memos

R4: You take the medicine and give to the child, however, the child would have been poorly treated, and the malaria then remains in the body and affects him/her. So the more you poorly treat the malaria, the more it sucks blood from the child and you will see the child's health situation worsening steadily. I have ever had a sick child and I took her for treatment in these small clinics but the child's health situation worsened steadily and yet the nurse could not tell me whether she will manage to treat the child's condition or not. She told me that we should put the child on a drip which she was also not aware of whether it will be helpful in treating my child or not until when my fellow women referred me to Masaka referral hospital. I went back to this nurse and told her to write for me a note indicating the medicine that she has been giving to my child so that I can take my child to Masaka. On reaching the hospital, they discovered that the child had a severe anemia and yet this Nurse had told me nothing relating to this; but just went on with her treatment.

P21: FGD_Severe_Anaemia_Masaka_01.rtf - 21:16 [R4: The failure to finish the ..]  (187:187)   (Super)
Codes:	[FGD 4.1 Causes of SA in children - Family: FGD 4.0 Causes of SA] [FGD 4.5 Processes leading to SA - Family: FGD 4.0 Causes of SA] 
No memos

R4: The failure to finish the dose is what we consider as the poorly treated malaria. You might choose to buy medicine from a drug-shop, start the dose but then fail to finish it. Children must be given 6 tabs of Coartem but one may first give the child 3 tabs but when they see that there is no change in the child's condition, they will change the child's treatment and when it also fails out they again change treatment instead of finishing the first dose of Coartem which they had earlier started hence leading to poorly treated malaria.

P21: FGD_Severe_Anaemia_Masaka_01.rtf - 21:17 [F: How does sickle cell lead t..]  (200:202)   (Super)
Codes:	[FGD 4.2 Blood sucking agents - Family: FGD 4.0 Causes of SA] [FGD 4.5 Processes leading to SA - Family: FGD 4.0 Causes of SA] 
No memos

F: How does sickle cell lead to severe anemia? What is sickle cells?
R5: One of my fellow respondents explained sickle cells as a recurrent disease to imply that it doesn't heal but continuously leads to severe anemia.
R2: it is like parasite feeding on someone daily.

P21: FGD_Severe_Anaemia_Masaka_01.rtf - 21:22 [F: How does an enlarged spleen..]  (235:236)   (Super)
Codes:	[FGD 4.2 Blood sucking agents - Family: FGD 4.0 Causes of SA] [FGD 4.5 Processes leading to SA - Family: FGD 4.0 Causes of SA] 
No memos

F: How does an enlarged spleen suck blood?
R1: I hear it is positioned near the heart from where it sucks blood.

P21: FGD_Severe_Anaemia_Masaka_01.rtf - 21:23 [R1: yes, I hear the “Male enla..]  (238:244)   (Super)
Codes:	[FGD 4.4 Enlarged spleen - Family: FGD 4.0 Causes of SA] [FGD 4.5 Processes leading to SA - Family: FGD 4.0 Causes of SA] 
No memos

R1: yes, I hear the “Male enlarged spleen” (“akasajja”) rests at the upper part of the abdomen while the female one (“akakazzi”) on the lower part of the abdomen.
F: What is do you mean by enlarged spleen?
R1: the doctors tell us that is a type of fever and that they have its medicine.
F: Is it found in the stomach?
All Rs: Yes
F: What does it look like?
R3: When you touch the child in the child's tummy you can feel it, it is hard but when you take the child for treatment and they cut it and put medicine, the child will be healed of it.

P22: FGD_Severe_Anaemia_Masaka_02.rtf - 22:8 [F: What makes you feel that th..]  (127:128)   (Super)
Codes:	[FGD 4.5 Processes leading to SA - Family: FGD 4.0 Causes of SA] 
No memos

F: What makes you feel that they are feeding poorly?
R3: Like young children eating plain cassava, drinking un-boiled water and then add on poor feeding and yet young.

P22: FGD_Severe_Anaemia_Masaka_02.rtf - 22:9 [R5: Now you find that the chil..]  (130:130)   (Super)
Codes:	[FGD 4.5 Processes leading to SA - Family: FGD 4.0 Causes of SA] 
No memos

R5: Now you find that the child is still young and have not even started crawling and they are feeding him on cassava, sometimes does not get food in time and yet doesn't breast feed in time.

P22: FGD_Severe_Anaemia_Masaka_02.rtf - 22:10 [R2: Me I think that poor feedi..]  (133:133)   (Super)
Codes:	[FGD 4.5 Processes leading to SA - Family: FGD 4.0 Causes of SA] 
No memos

R2: Me I think that poor feeding is when you give the child meals that do not match his body. There are foods which he doesn't get yet his body needs them

P22: FGD_Severe_Anaemia_Masaka_02.rtf - 22:11 [: Say what you know or what yo..]  (138:140)   (Super)
Codes:	[FGD 4.1 Causes of SA in children - Family: FGD 4.0 Causes of SA] [FGD 4.5 Processes leading to SA - Family: FGD 4.0 Causes of SA] 
No memos

: Say what you know or what you hear.
R5; Even poverty causes SA.
R1; poor health care. The child may suffer from malaria and the parent doesn't care and says aaa that's how he is always. So the malaria becomes severe.

P22: FGD_Severe_Anaemia_Masaka_02.rtf - 22:12 [F: How does the malaria cause ..]  (147:148)   (Super)
Codes:	[FGD 4.5 Processes leading to SA - Family: FGD 4.0 Causes of SA] 
No memos

F: How does the malaria cause severe anemia in a child?
R3: Now doctor, a child might suffer malaria and the parent becomes negligent and don't take the child to the hospital thinking the child will be fine. She prepares some herbal medicine and doesn't confirm whether the child is healed. Then after some time the child becomes sick again. Eventually you hear that the child has no blood, no water.

FGD 4.6 Causes of SA_ranked
______________________________________________________________________

P18: FGD_Severe_Anaemia_Hoima_02.rtf - 18:7 [SA; Today we are talking about..]  (58:59)   (Super)
Codes:	[FGD 4.6 Causes of SA_ranked - Family: FGD 4.0 Causes of SA] 
No memos

SA; Today we are talking about only severe anemia..mmmm.. Now according to your thinking, what do you think causes severe anemia in children?
R3. I think it's due to persistent and recurrent malaria because if it finishes like one week and it comes back… that can cause severe anemia..mmm…

P21: FGD_Severe_Anaemia_Masaka_01.rtf - 21:27 [F: Let us agree, which one is ..]  (353:371)   (Super)
Codes:	[FGD 4.6 Causes of SA_ranked - Family: FGD 4.0 Causes of SA] 
No memos

F: Let us agree, which one is the most common among the causes that you mentioned?
R4: Delay in giving the child treatment
R3: Yes, the delay in treatment / in seeking care
F: “eeh,” failure to treat the child early, then the 2nd one?
F: What about the tape worms?
F: What about these things of ours (evil spirits) you mean they don't lead to severe anemia at a high rate?
R3: Do you mean “ebijinijini?”
R1: They also cause severe anemia because it's the purpose (to suck blood) as to why people send them.
F: Yes, I have requested that we rank them.
R1: Yes, the evil spirits although suck blood, they are not as common as the other causes.
R3: Malaria fever is the most common cause of severe anemia.
R1: I think there is no need to consider sickle cells since it has already been considered under malaria fever.
F: What about the enlarged spleen?
R6: It also causes severe anemia.
R3: And it sucks blood at a very high rate to the extent that the child doesn't remain with any blood.
F: Between the tape worms and enlarged spleen which one takes the 3rd position?
All respondents: the enlarged spleen
R3: That one leads to complete severe anemia. Even though you are an adult, they will find you with no blood at all.
F: Poor feeding, poor treatment and the enlarged spleen, those are the 3 most common causes of severe lack of blood.

P22: FGD_Severe_Anaemia_Masaka_02.rtf - 22:17 [F: We have talked all those an..]  (207:213)   (Super)
Codes:	[FGD 4.6 Causes of SA_ranked - Family: FGD 4.0 Causes of SA] 
No memos

F: We have talked all those and you had even forgotten some. But rank for me the commonest three that suck blood from children.
R4: Poor feeding
F: Which the second one?
All Rs: enlarged spleen
R3: AIDS 
F: So the three are the worst?
R3: Yes according to our thinking. (…….Laughs)


Code Family: FGD 5.0 Care seeking for children with SA
______________________________________________________________________

HU:	Local Perceptions of Childhood Severe Anaemia_FA_January 2018
File:	 [C:\Users\Florence\Desktop\Local Perceptions of Childhood Severe Anaemia_FA_January 2018.hpr7]
Edited by:	Super
Date/Time:	2018-01-05 10:04:01
______________________________________________________________________

Created: 2017-12-18 09:44:03 (Super) 
Codes (4):	[FGD 5.1 Health care seeking behaviour patterns for SA] [FGD 5.2 Reasons for health care seeking behav pttns for SA] [FGD 5.3 Gender roles in the care of Chldn with SA] [FGD 5.4 Decision making in the care of Chldn with SA]
Quotation(s): 18
______________________________________________________________________

FGD 5.1 Health care seeking behaviour patterns for SA
______________________________________________________________________

P 1: IDI_Severe_Anaemia_Jinja_001.rtf - 1:13 [Interviewer: How have you been..]  (122:125)   (Super)
Codes:	[FGD 5.1 Health care seeking behaviour patterns for SA - Family: FGD 5.0 Care seeking for children with SA] [FGD 5.2 Reasons for health care seeking behav pttns for SA - Family: FGD 5.0 Care seeking for children with SA] 
No memos

Interviewer: How have you been managing the children who get severe anemia before reaching the hospital. What treatment do you give your children with severe anemia?
Respondent: Now these children, the treatment i have been giving them, like you know you go to some clinics and buy some tablets if they get malaria then they improve a bit but if it is something to do with severe anemia, it is impossible. now what can work for this child is this hospital because there is nowhere you will go in the village to buy tablets to give her to improve. 
Interviewer: That means the moment you notice, you seek medical help?
Respondent: I jump onto the ferry, and i cross the lake to come here and i tell them your client is here and they start treating....and they even say, again muzee you are back?... that i am back. if i stay there and i delay, they will say why did you not come early..so i bring her when she is still a bit strong and they begin from there.

P17: FGD_Severe_Anaemia_Hoima_01.rtf - 17:15 [SA; There are others who told ..]  (76:78)   (Super)
Codes:	[FGD 5.1 Health care seeking behaviour patterns for SA - Family: FGD 5.0 Care seeking for children with SA] 
No memos

SA; There are others who told us about evils spirits and witchcraft sucking blood, for you what do you think?
Res; for us we know its only sickness.
Res; for us we take them to the hospital. we don't go to witch doctors. Can a witch doctor inject you? You have to go to the doctor and he tells you what to do, the rest you leave God to decide

P17: FGD_Severe_Anaemia_Hoima_01.rtf - 17:16 [SA; What I wanted to know; the..]  (79:81)   (Super)
Codes:	[FGD 4.3 Evil spirits - Family: FGD 4.0 Causes of SA] [FGD 5.1 Health care seeking behaviour patterns for SA - Family: FGD 5.0 Care seeking for children with SA] 
No memos

SA; What I wanted to know; there are other people who say that the evil spirits and witch craft suck blood. What do you know about this?
Res; we hear that from other people. That it is Satan and then they go to prayer warriors yet the child has severe anemia…mm… or they remain at home using home remedies and finally the child dies. 
Res; when a child becomes sick everyone talks to his mind, others that the child is bewitched; take the child for prayers but for me I decide to take mine to the hospital….mmm…

P17: FGD_Severe_Anaemia_Hoima_01.rtf - 17:17 [SA; How do you treat severe an..]  (82:85)   (Super)
Codes:	[FGD 5.1 Health care seeking behaviour patterns for SA - Family: FGD 5.0 Care seeking for children with SA] [FGD 5.2 Reasons for health care seeking behav pttns for SA - Family: FGD 5.0 Care seeking for children with SA] 
No memos

SA; How do you treat severe anemia; what do you do to a child with severe anemia?
R1; for me my child became sick in June and I took him to kigorobya health center. It is a government hospital. But I took him there when he was not very badly off. But when I reached to the doctor he told me that the child has no enough blood he prescribed for me drugs that restore blood. He also told me to cook for him greens and the child will normalize…mmm…
SA; which drugs did the doctor give you?
R1; He gave me the drug called FerroB

P18: FGD_Severe_Anaemia_Hoima_02.rtf - 18:18 [SA; what do you do to a child ..]  (120:121)   (Super)
Codes:	[FGD 5.1 Health care seeking behaviour patterns for SA - Family: FGD 5.0 Care seeking for children with SA] [FGD 5.2 Reasons for health care seeking behav pttns for SA - Family: FGD 5.0 Care seeking for children with SA] 
No memos

SA; what do you do to a child who has severe anemia?
RES; When a child becomes sick and you realize that probably she is experiencing severe anemia, you rash her to the hospital….mmmm.. You take him to the hospital and they also device means of treatment if blood is not available they refer you to another hospital where blood transfusion can be done...mmm..

P21: FGD_Severe_Anaemia_Masaka_01.rtf - 21:11 [F: In your community, do paren..]  (156:157)   (Super)
Codes:	[FGD 5.1 Health care seeking behaviour patterns for SA - Family: FGD 5.0 Care seeking for children with SA] [FGD 5.2 Reasons for health care seeking behav pttns for SA - Family: FGD 5.0 Care seeking for children with SA] 
No memos

F: In your community, do parents usually treat their children from home without taking them to the hospital?
R3: Some parents it could be that they have no money and may just go and pick some medicine.

P21: FGD_Severe_Anaemia_Masaka_01.rtf - 21:28 [F: what do you do for your chi..]  (373:378)   (Super)
Codes:	[FGD 5.1 Health care seeking behaviour patterns for SA - Family: FGD 5.0 Care seeking for children with SA] 
No memos

F: what do you do for your children with severe anemia?
R3: I take them to Masaka Regional R. hospital and they put blood on them. They put blood drip on the child and also give him or her some tabs hence recovering.
F: Apart from Masaka referral hospital, is there any other place where they carry out a blood transfusion?
R3: You have to go to either Masaka referral hospital or Kitovu hospital
R2: those are the only places that I also know 
R4: you go to Dr. Kasozi for the blood transfusion.

P21: FGD_Severe_Anaemia_Masaka_01.rtf - 21:32 [F: So what happens when the si..]  (406:407)   (Super)
Codes:	[FGD 5.1 Health care seeking behaviour patterns for SA - Family: FGD 5.0 Care seeking for children with SA] [FGD 5.2 Reasons for health care seeking behav pttns for SA - Family: FGD 5.0 Care seeking for children with SA] 
No memos

F: So what happens when the sickness is mild?
R3: you cook for them the local herbs and you will realize later on that they have improved.

P22: FGD_Severe_Anaemia_Masaka_02.rtf - 22:20 [F: Apart from that, is there a..]  (260:263)   (Super)
Codes:	[FGD 5.1 Health care seeking behaviour patterns for SA - Family: FGD 5.0 Care seeking for children with SA] [FGD 5.2 Reasons for health care seeking behav pttns for SA - Family: FGD 5.0 Care seeking for children with SA] 
No memos

F: Apart from that, is there any other treatment you know of for severe anemia?
R7: to take him to the hospital.
R7: the other one (the herbs) is like the first aid.
R2: even in the hospital if they put him on a drip it may not work and the doctors advise you to look for herbal medicine.

FGD 5.2 Reasons for health care seeking behav pttns for SA
______________________________________________________________________

P 1: IDI_Severe_Anaemia_Jinja_001.rtf - 1:13 [Interviewer: How have you been..]  (122:125)   (Super)
Codes:	[FGD 5.1 Health care seeking behaviour patterns for SA - Family: FGD 5.0 Care seeking for children with SA] [FGD 5.2 Reasons for health care seeking behav pttns for SA - Family: FGD 5.0 Care seeking for children with SA] 
No memos

Interviewer: How have you been managing the children who get severe anemia before reaching the hospital. What treatment do you give your children with severe anemia?
Respondent: Now these children, the treatment i have been giving them, like you know you go to some clinics and buy some tablets if they get malaria then they improve a bit but if it is something to do with severe anemia, it is impossible. now what can work for this child is this hospital because there is nowhere you will go in the village to buy tablets to give her to improve. 
Interviewer: That means the moment you notice, you seek medical help?
Respondent: I jump onto the ferry, and i cross the lake to come here and i tell them your client is here and they start treating....and they even say, again muzee you are back?... that i am back. if i stay there and i delay, they will say why did you not come early..so i bring her when she is still a bit strong and they begin from there.

P17: FGD_Severe_Anaemia_Hoima_01.rtf - 17:17 [SA; How do you treat severe an..]  (82:85)   (Super)
Codes:	[FGD 5.1 Health care seeking behaviour patterns for SA - Family: FGD 5.0 Care seeking for children with SA] [FGD 5.2 Reasons for health care seeking behav pttns for SA - Family: FGD 5.0 Care seeking for children with SA] 
No memos

SA; How do you treat severe anemia; what do you do to a child with severe anemia?
R1; for me my child became sick in June and I took him to kigorobya health center. It is a government hospital. But I took him there when he was not very badly off. But when I reached to the doctor he told me that the child has no enough blood he prescribed for me drugs that restore blood. He also told me to cook for him greens and the child will normalize…mmm…
SA; which drugs did the doctor give you?
R1; He gave me the drug called FerroB

P18: FGD_Severe_Anaemia_Hoima_02.rtf - 18:18 [SA; what do you do to a child ..]  (120:121)   (Super)
Codes:	[FGD 5.1 Health care seeking behaviour patterns for SA - Family: FGD 5.0 Care seeking for children with SA] [FGD 5.2 Reasons for health care seeking behav pttns for SA - Family: FGD 5.0 Care seeking for children with SA] 
No memos

SA; what do you do to a child who has severe anemia?
RES; When a child becomes sick and you realize that probably she is experiencing severe anemia, you rash her to the hospital….mmmm.. You take him to the hospital and they also device means of treatment if blood is not available they refer you to another hospital where blood transfusion can be done...mmm..

P21: FGD_Severe_Anaemia_Masaka_01.rtf - 21:11 [F: In your community, do paren..]  (156:157)   (Super)
Codes:	[FGD 5.1 Health care seeking behaviour patterns for SA - Family: FGD 5.0 Care seeking for children with SA] [FGD 5.2 Reasons for health care seeking behav pttns for SA - Family: FGD 5.0 Care seeking for children with SA] 
No memos

F: In your community, do parents usually treat their children from home without taking them to the hospital?
R3: Some parents it could be that they have no money and may just go and pick some medicine.

P21: FGD_Severe_Anaemia_Masaka_01.rtf - 21:32 [F: So what happens when the si..]  (406:407)   (Super)
Codes:	[FGD 5.1 Health care seeking behaviour patterns for SA - Family: FGD 5.0 Care seeking for children with SA] [FGD 5.2 Reasons for health care seeking behav pttns for SA - Family: FGD 5.0 Care seeking for children with SA] 
No memos

F: So what happens when the sickness is mild?
R3: you cook for them the local herbs and you will realize later on that they have improved.

P22: FGD_Severe_Anaemia_Masaka_02.rtf - 22:20 [F: Apart from that, is there a..]  (260:263)   (Super)
Codes:	[FGD 5.1 Health care seeking behaviour patterns for SA - Family: FGD 5.0 Care seeking for children with SA] [FGD 5.2 Reasons for health care seeking behav pttns for SA - Family: FGD 5.0 Care seeking for children with SA] 
No memos

F: Apart from that, is there any other treatment you know of for severe anemia?
R7: to take him to the hospital.
R7: the other one (the herbs) is like the first aid.
R2: even in the hospital if they put him on a drip it may not work and the doctors advise you to look for herbal medicine.

FGD 5.3 Gender roles in the care of Chldn with SA
______________________________________________________________________

P19: FGD_Severe_Anaemia_Jinja_01.rtf - 19:32 [Moderator: wait…we are still d..]  (337:340)   (Super)
Codes:	[FGD 5.3 Gender roles in the care of Chldn with SA - Family: FGD 5.0 Care seeking for children with SA] [FGD 5.4 Decision making in the care of Chldn with SA - Family: FGD 5.0 Care seeking for children with SA] 
No memos

Moderator: wait…we are still discussing………now when we are at home for example husband, wife the children; boys and girls and we get a problem of severe anemia with one of the children?....who does what with regard to responsibilities…….
Respondents: it's the mother……..
Moderator: who does what? What does the mother do and what does the father do?.......Annet, you hard started explaining……..
Respondents: (in chorus) hmhm……….it is me the mother who takes over……the father can be there and say….this child, go and boil for him some herbs…..and you go and cook……He may not even be around or he can wake up in the morning and say…'the child is sick but you will see what to do”……he goes away and you have to see what to do for the child…by the time he comes back, you have cared for the child and the child is fine not like he left him…….but Musawo, these days men do not have a sense of responsibility…..for him…..it is you (the mother) who cares about your child……as long as long as you tell him that the child is sick….he will tell you take him to hospital…..ahaaa….others say now you are telling me. am I a hospital?

P19: FGD_Severe_Anaemia_Jinja_01.rtf - 19:33 [Respondent: he can leave you t..]  (346:346)   (Super)
Codes:	[FGD 5.3 Gender roles in the care of Chldn with SA - Family: FGD 5.0 Care seeking for children with SA] [FGD 5.4 Decision making in the care of Chldn with SA - Family: FGD 5.0 Care seeking for children with SA] 
No memos

Respondent: he can leave you two thousand for transport and he says “you will see what to do with your child”….go and buy panadol….whether you go to see musawo, or to the hospital….its up to you………the rest you will call me when you come back……you come back with your receipt then he will say…but will we get the money?........do you still wait for the money musawo? You have to use your brain like any other woman does….like the way we are us women and plan………………….

P20: FGD_Severe_Anaemia_Jinja_02.rtf - 20:30 [Moderator: okay lets continue…..]  (247:248)   (Super)
Codes:	[FGD 5.3 Gender roles in the care of Chldn with SA - Family: FGD 5.0 Care seeking for children with SA] 
No memos

Moderator: okay lets continue…… take for instance at home you are mother, father, children (male and female) and one child looses blood, who does what in this case, or who plays what role?
Respondents: it's the mother that takes responsibility

P20: FGD_Severe_Anaemia_Jinja_02.rtf - 20:31 [Respondents: mothers take resp..]  (250:253)   (Super)
Codes:	[FGD 5.3 Gender roles in the care of Chldn with SA - Family: FGD 5.0 Care seeking for children with SA] 
No memos

Respondents: mothers take responsibility in looking for the local herbs and if the herbs fail, now the father comes in to take to the hospital or in most cases he just gives money 
Moderator: Meaning they all play a role right….?
Respondents: yes and even when the father is not around, the elder son works hard to bring a bodaboda to take the child to the hospital. Meaning that anyone around the sick child takes responsibility 
Moderator: you said fathers just gives money …

P21: FGD_Severe_Anaemia_Masaka_01.rtf - 21:34 [F: As a family, while taking c..]  (433:437)   (Super)
Codes:	[FGD 5.3 Gender roles in the care of Chldn with SA - Family: FGD 5.0 Care seeking for children with SA] [FGD 5.4 Decision making in the care of Chldn with SA - Family: FGD 5.0 Care seeking for children with SA] 
No memos

F: As a family, while taking care of a child with severe anemia, is it only the woman who plays a role or there is sharing of responsibilities?
R6: it is mainly the women that take care of the children, the men are not usually around. They only contribute financially.
All respondents: it's the woman.
R1: many times the men do not care at all.
R6: it is the women that take care 

P21: FGD_Severe_Anaemia_Masaka_01.rtf - 21:36 [F: Apart from money, is there ..]  (442:446)   (Super)
Codes:	[FGD 5.3 Gender roles in the care of Chldn with SA - Family: FGD 5.0 Care seeking for children with SA] 
No memos

F: Apart from money, is there any other way in which the men care for their children with severe anemia?
R1: Not at all, it is you (the woman) that suffers with your child and will narrate him what you went through when you get back home.  
R4: I could be in the village right now and see that the child has severe anemia and call him and tell him to go to Nyendo to help me buy it because it is over, therefore, he will go and buy it.
F: It is the woman that has to strive for her child?
All Rs: Yes

P22: FGD_Severe_Anaemia_Masaka_02.rtf - 22:21 [F: Let me take you back for th..]  (264:272)   (Super)
Codes:	[FGD 5.3 Gender roles in the care of Chldn with SA - Family: FGD 5.0 Care seeking for children with SA] 
No memos

F: Let me take you back for the treatment of a child with severe anemia, how do you share the responsibilities if you're at home; mother and father?
R7: I do them (giving herbs) in his absence because even if he is there you can tell him that a child has lost blood and asks you where you have measured him from.
F: You do it yourself and do what you know?
R2: Yes.
R1: Let me also ask a question; if a mother or wife are not at home and the child loses blood what can be done?
F: Me I wanted a mother and father?
R3: for the father when you tell him that a child has lost blood, automatically he asks where you have measured it from, have you tested him, are you a doctor?
R3: When you start explaining to him, he will tell you to look local herbs.
R3: When you tell him you don't know he will refer you to ask your fellow women.

FGD 5.4 Decision making in the care of Chldn with SA
______________________________________________________________________

P19: FGD_Severe_Anaemia_Jinja_01.rtf - 19:32 [Moderator: wait…we are still d..]  (337:340)   (Super)
Codes:	[FGD 5.3 Gender roles in the care of Chldn with SA - Family: FGD 5.0 Care seeking for children with SA] [FGD 5.4 Decision making in the care of Chldn with SA - Family: FGD 5.0 Care seeking for children with SA] 
No memos

Moderator: wait…we are still discussing………now when we are at home for example husband, wife the children; boys and girls and we get a problem of severe anemia with one of the children?....who does what with regard to responsibilities…….
Respondents: it's the mother……..
Moderator: who does what? What does the mother do and what does the father do?.......Annet, you hard started explaining……..
Respondents: (in chorus) hmhm……….it is me the mother who takes over……the father can be there and say….this child, go and boil for him some herbs…..and you go and cook……He may not even be around or he can wake up in the morning and say…'the child is sick but you will see what to do”……he goes away and you have to see what to do for the child…by the time he comes back, you have cared for the child and the child is fine not like he left him…….but Musawo, these days men do not have a sense of responsibility…..for him…..it is you (the mother) who cares about your child……as long as long as you tell him that the child is sick….he will tell you take him to hospital…..ahaaa….others say now you are telling me. am I a hospital?

P19: FGD_Severe_Anaemia_Jinja_01.rtf - 19:33 [Respondent: he can leave you t..]  (346:346)   (Super)
Codes:	[FGD 5.3 Gender roles in the care of Chldn with SA - Family: FGD 5.0 Care seeking for children with SA] [FGD 5.4 Decision making in the care of Chldn with SA - Family: FGD 5.0 Care seeking for children with SA] 
No memos

Respondent: he can leave you two thousand for transport and he says “you will see what to do with your child”….go and buy panadol….whether you go to see musawo, or to the hospital….its up to you………the rest you will call me when you come back……you come back with your receipt then he will say…but will we get the money?........do you still wait for the money musawo? You have to use your brain like any other woman does….like the way we are us women and plan………………….

P20: FGD_Severe_Anaemia_Jinja_02.rtf - 20:32 [Moderator: you have talked abo..]  (257:260)   (Super)
Codes:	[FGD 5.4 Decision making in the care of Chldn with SA - Family: FGD 5.0 Care seeking for children with SA] 
No memos

Moderator: you have talked about responsibilities, who then decides that we give herbals or take to hospital?
Respondents: hmhmmmmm,…. It depends on the severity of the sickness but the decision is taken by both man and woman and after trying all herbs in vain..... I call him and tell him herbs have failed because it is me that knows the state of the child after trying all the local herbs and when he is not at home I call him using a phone and ask him to find me at the hospital.
Moderator: you mean decision to use herbal is for a women and hospital is for a man?
Respondents: No, the decisions are two way, we both play a role because there some women who are lazy and not active to take action on what to do. So in this case a man asks her to get herbals but where both are active they work together. 

P21: FGD_Severe_Anaemia_Masaka_01.rtf - 21:34 [F: As a family, while taking c..]  (433:437)   (Super)
Codes:	[FGD 5.3 Gender roles in the care of Chldn with SA - Family: FGD 5.0 Care seeking for children with SA] [FGD 5.4 Decision making in the care of Chldn with SA - Family: FGD 5.0 Care seeking for children with SA] 
No memos

F: As a family, while taking care of a child with severe anemia, is it only the woman who plays a role or there is sharing of responsibilities?
R6: it is mainly the women that take care of the children, the men are not usually around. They only contribute financially.
All respondents: it's the woman.
R1: many times the men do not care at all.
R6: it is the women that take care 

P21: FGD_Severe_Anaemia_Masaka_01.rtf - 21:35 [F: One of you has mentioned th..]  (438:441)   (Super)
Codes:	[FGD 5.4 Decision making in the care of Chldn with SA - Family: FGD 5.0 Care seeking for children with SA] 
No memos

F: One of you has mentioned that men usually leave some money for medication, what role is played by men when it comes to taking care of a child with severe anemia?
R2: That is what they usually stop at.
R3: If they have helped you much, they will give you some money for medication. Thereafter, you will seek the medication by yourself.
R5: when he sees you not return back home, you then call him and tell him that you have been admitted. However, they offer financial help.
